# MOG-IgG in NMO and related disorders: a multicenter study of 50 patients. Part 2: Epidemiology, clinical presentation, radiological and laboratory features, treatment responses, and long-term outcome

**DOI:** 10.1186/s12974-016-0718-0

**Published:** 2016-09-27

**Authors:** Sven Jarius, Klemens Ruprecht, Ingo Kleiter, Nadja Borisow, Nasrin Asgari, Kalliopi Pitarokoili, Florence Pache, Oliver Stich, Lena-Alexandra Beume, Martin W. Hümmert, Marius Ringelstein, Corinna Trebst, Alexander Winkelmann, Alexander Schwarz, Mathias Buttmann, Hanna Zimmermann, Joseph Kuchling, Diego Franciotta, Marco Capobianco, Eberhard Siebert, Carsten Lukas, Mirjam Korporal-Kuhnke, Jürgen Haas, Kai Fechner, Alexander U. Brandt, Kathrin Schanda, Orhan Aktas, Friedemann Paul, Markus Reindl, Brigitte Wildemann, in cooperation with the Neuromyelitis Optica Study Group (NEMOS)

**Affiliations:** 1 Molecular Neuroimmunology Group, Department of Neurology, University of Heidelberg, Heidelberg, Germany; 2 Department of Neurology, Charité University Medicine Berlin, Berlin, Germany; 3 Department of Neurology, Ruhr University Bochum, Bochum, Germany; 4 NeuroCure Clinical Research Center and Clinical and Experimental Multiple Sclerosis Research Center, Department of Neurology, Charité University Medicine, Berlin, Germany; 5 Experimental and Clinical Research Center, Max Delbrueck Center for Molecular Medicine and Charité University Medicine Berlin, Berlin, Germany; 6 Department of Neurology and Institute of Molecular Medicine, University of Southern Denmark, Odense, Denmark; 7 Department of Neurology, Albert Ludwigs University, Freiburg, Germany; 8 Department of Neurology, Hannover Medical School, Hannover, Germany; 9 Department of Neurology, Heinrich Heine University, Düsseldorf, Germany; 10 Department of Neurology, University of Rostock, Rostock, Germany; 11 Department of Neurology, Julius Maximilians University, Würzburg, Germany; 12 IRCCS, C. Mondino National Neurological Institute, Pavia, Italy; 13 Centro di Riferimento Regionale SM, Azienda Ospedaliero Universitaria San Luigi Gonzaga, Orbassano, Italy; 14 Department of Neuroradiology, Charité University Medicine – Berlin, Berlin, Germany; 15 Department of Neuroradiology, Ruhr University Bochum, Bochum, Germany; 16 Institute of Experimental Immunolog, affiliated to Euroimmun AG, Lübeck, Germany; 17 Department of Neurology, Medical University Innsbruck, Innsbruck, Austria

**Keywords:** Myelin oligodendrocyte glycoprotein antibodies (MOG-IgG), Autoantibodies, Neuromyelitis optica spectrum disorders (NMOSD), Aquaporin-4 antibodies (AQP4-IgG, NMO-IgG), Optic neuritis, Transverse myelitis, Longitudinally extensive transverse myelitis, Magnetic resonance imaging, Cerebrospinal fluid, Oligoclonal bands, Electrophysiology, Evoked potentials, Treatment, Therapy, Methotrexate, Azathioprine, Rituximab, Ofatumumab, Interferon beta, Glatiramer acetate, Natalizumab, Outcome, Pregnancy, Infections, Vaccination, Multiple sclerosis, Barkhof criteria, McDonald criteria, Wingerchuk criteria 2006 and 2015, IPND criteria, International consensus diagnostic criteria for neuromyelitis optica spectrum disorders

## Abstract

**Background:**

A subset of patients with neuromyelitis optica spectrum disorders (NMOSD) has been shown to be seropositive for myelin oligodendrocyte glycoprotein antibodies (MOG-IgG).

**Objective:**

To describe the epidemiological, clinical, radiological, cerebrospinal fluid (CSF), and electrophysiological features of a large cohort of MOG-IgG-positive patients with optic neuritis (ON) and/or myelitis (*n* = 50) as well as attack and long-term treatment outcomes.

**Methods:**

Retrospective multicenter study.

**Results:**

The sex ratio was 1:2.8 (m:f). Median age at onset was 31 years (range 6-70). The disease followed a multiphasic course in 80 % (median time-to-first-relapse 5 months; annualized relapse rate 0.92) and resulted in significant disability in 40 % (mean follow-up 75 ± 46.5 months), with severe visual impairment or functional blindness (36 %) and markedly impaired ambulation due to paresis or ataxia (25 %) as the most common long-term sequelae. Functional blindess in one or both eyes was noted during at least one ON attack in around 70 %. Perioptic enhancement was present in several patients. Besides acute tetra-/paraparesis, dysesthesia and pain were common in acute myelitis (70 %). Longitudinally extensive spinal cord lesions were frequent, but short lesions occurred at least once in 44 %. Fourty-one percent had a history of simultaneous ON and myelitis. Clinical or radiological involvement of the brain, brainstem, or cerebellum was present in 50 %; extra-opticospinal symptoms included intractable nausea and vomiting and respiratory insufficiency (fatal in one). CSF pleocytosis (partly neutrophilic) was present in 70 %, oligoclonal bands in only 13 %, and blood-CSF-barrier dysfunction in 32 %. Intravenous methylprednisolone (IVMP) and long-term immunosuppression were often effective; however, treatment failure leading to rapid accumulation of disability was noted in many patients as well as flare-ups after steroid withdrawal. Full recovery was achieved by plasma exchange in some cases, including after IVMP failure. Breakthrough attacks under azathioprine were linked to the drug-specific latency period and a lack of cotreatment with oral steroids. Methotrexate was effective in 5/6 patients. Interferon-beta was associated with ongoing or increasing disease activity. Rituximab and ofatumumab were effective in some patients. However, treatment with rituximab was followed by early relapses in several cases; end-of-dose relapses occurred 9-12 months after the first infusion. Coexisting autoimmunity was rare (9 %). Wingerchuk’s 2006 and 2015 criteria for NMO(SD) and Barkhof and McDonald criteria for multiple sclerosis (MS) were met by 28 %, 32 %, 15 %, 33 %, respectively; MS had been suspected in 36 %. Disease onset or relapses were preceded by infection, vaccination, or pregnancy/delivery in several cases.

**Conclusion:**

Our findings from a predominantly Caucasian cohort strongly argue against the concept of MOG-IgG denoting a mild and usually monophasic variant of NMOSD. The predominantly relapsing and often severe disease course and the short median time to second attack support the use of prophylactic long-term treatments in patients with MOG-IgG-positive ON and/or myelitis.

## Background

The term ‘neuromyelitis optica’ (NMO) was coined in 1894 and has since been used to refer to the simultaneous or successive occurrence of optic nerve and spinal cord inflammation [1]. In the majority of cases, the syndrome is caused by autoantibodies to aquaporin-4, the most common water channel in the central nervous system (AQP4-IgG) [2–5]. However, 10-20 % of patients with NMO are negative for AQP4-IgG [6–9]. Recent studies by us and others have demonstrated the presence of IgG antibodies to myelin oligodendrocyte glycoprotein (MOG-IgG) in a subset of patients with NMO as well as in patients with isolated ON or longitudinally extensive transverse myelitis (LETM), syndromes that are often *formes frustes* of NMO [10–12].

Most studies to date have found MOG-IgG exclusively in AQP4-IgG-negative patients [11–17]. Moreover, the histopathology of brain and spinal cord lesions of MOG-IgG-positive patients has been shown to differ from that of AQP4-IgG-posititve patients [18–20]. Finally, evidence from immunological studies suggests a direct pathogenic role of MOG-IgG both in vitro and in vivo [10, 21]. Accordingly, MOG-IgG-related NMO is now considered by many as a disease entity in its own right, immunopathogenetically distinct from its AQP4-IgG-positive counterpart. However, the cohorts included in previous clinical studies were relatively small (median 9 patients in [10–17, 22–24]) and the observation periods often short (median 24 months in [11–13, 15–17, 23–26]). Moreover, some previous studies did not, or not predominantly, include Caucasian patients [12, 15, 26], which is potentially important since genetic factors are thought to play a role in NMO [27].

In the present study, we systematically evaluated the clinical and paraclinical features of a large cohort of 50 almost exclusively Caucasian patients with MOG-IgG-positive optic neuritis (ON) and/or LETM. We report on (i) epidemiological features; (ii) clinical presentation at onset; (iii) disease course; (iv) time to second attack; (v) type and frequency of clinical attacks; (vi) brain, optic nerve, and spinal cord magnetic resonance imaging (MRI) features; (vii) cerebrospinal fluid (CSF) findings; (viii) electrophysiological features (VEP, SSEP); (ix) type and frequency of coexisting autoimmunity; (x) type and frequency of preceding infections; (xi) association with neoplasms; (xii) association with pregnancy and delivery; (xiii) treatment and outcome of acute attacks; (xiv) response to long-term treatments; and (xv) the long-term prognosis. In addition, we evaluated whether and how many MOG-IgG-positive patients with ON and/or myelitis met Wingerchuk’s revised 2006 diagnostic criteria for NMO [28], the new 2015 international diagnostic consensus criteria for NMO spectrum disorders (NMOSD) [29], Barkhof’s MRI criteria for MS, and/or McDonald’s clinicoradiological criteria for MS.

The present study forms part of a series of articles on MOG-IgG in NMO and related disorders. In part 1, we investigated the frequency and syndrome specificity of MOG-IgG among patients with ON and/or LETM, reported on MOG-IgG titers in the long-term course of disease, and analyzed the origin of CSF MOG-IgG [30]. In part 3, we describe in detail the clinical course and presentation of a subgroup of patients with brainstem encephalitis and MOG-IgG-associated ON and/or LETM, a so far under-recognized manifestation of MOG-related autoimmunity [31]. Part 4 is dedicated to the visual system in MOG-IgG-positive patients with ON and reports findings from optical coherence tomography (OCT) in this entity [32].

## Methods

Clinical and paraclinical data of 50 MOG-IgG-positive patients from 12 non-pediatric academic centers were retrospectively evaluated; eight of the participating centers are members of the German Neuromyelitis optica Study Group (NEMOS) [33–37]. MOG-IgG was detected using an in-house cell-based assay (CBA) employing HEK293A cells transfected with full-length human MOG as previously described [10] and confirmed by means of a commercial fixed-cell based assay employing HEK293 cells transfected with full-length human MOG (Euroimmun, Lübeck, Germany) (see part 1 of this article series for details [30]). The study was approved by the institutional review boards of the participating centers, and patients gave written informed consent. Averages are given as median and range or mean and standard deviation as indicated. Fisher’s exact test was used to compare frequencies between groups and the Mann-Whitney U test to compare medians between groups. Due to the exploratory nature of this study no Bonferroni correction was performed. *P* values <0.05 were considered statistically significant.

### Case reports

As reliable cell-based assays for the detection of MOG-IgG have become available only recently, large and comprehensive case series illustrating the broad and heterogeneous spectrum of clinical manifestations, disease courses, and radiological presentations are lacking so far. We therefore decided to present, in addition to descriptive statistical data, detailed reports on all cases evaluated in order to draw for the first time a more vivid ‘real-life’ picture of this rare disorder than statistical analyses alone could provide. Moreover, only detailed case descriptions allow evaluation of treatment responses and outcomes in a meaningful way in a retrospective setting. This is important, since randomized treatment trials in MOG-IgG-positive ON or myelitis do not exist so far and will not be performed in the near future due to the rarity of the condition. The reports are to be found in the Appendix of this paper and in the *Case reports* section in part 3 of this article series [31].

## Results

### Epidemiological findings

**Fig. 1:**
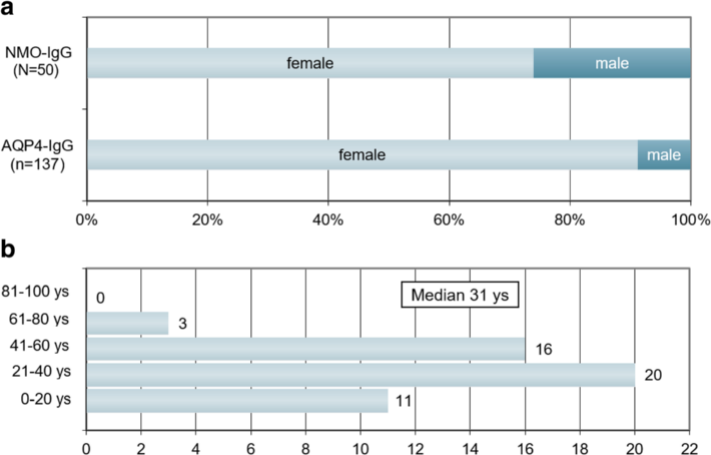
Sex ratio and age distribution. **a** Sex ratio in MOG-IgG-positive patients with ON and/or LETM compared with AQP4-IgG-positive ON and/or LETM (the latter data are taken from ref. [34]). **b** Age distribution at disease onset in 50 MOG-IgG-positive patients with ON and/or myelitis

Thirty-seven of the 50 MOG-IgG-positive patients were female, corresponding to a sex ratio of 1:2.8 (m:f) (Fig. 1a). Median age at onset was 31 years (35.5 years in patients presenting with isolated ON [*N* = 32] and 28.5 years in the remainder [*N* = 18]; *p* < 0.04) with a broad range of 6 to 70 years. 3 patients were > =60 years of age at onset, and 8 patients were under 18 at first attack (including 4 ≤ 12 years) (Fig. 1b). Fourty-nine of the 50 patients (98 %) were of Caucasian and 1 of Asian descent. Symptoms had started between Jul 1973 and Apr 2016. The mean observation period since disease onset was 75 ± 46.5 months (range 1-507 months). In line with the fact that many MOG-IgG-positive patients develop ON and myelitis only successively, the mean observation period was longer in patients with a history both of ON and of myelitis at last follow-up (88.6 months; *N* = 22) than in patients with a history of either ON but no myelitis or myelitis but not ON (64.6 months; *N* = 28).

### Disease course

**Fig. 2:**
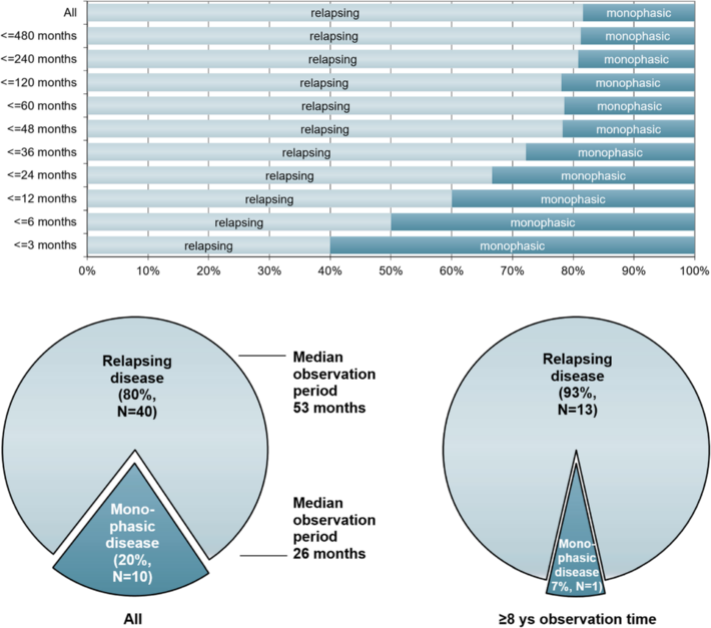
Disease course in relation to observation time in 50 MOG-IgG-positive patients with ON and/or myelitis. Upper panel: Note the decrease in the proportion of monophasic cases with increasing observation time; however, in some patients no relapse has occurred more than 10 years after the initial attack. Lower panel: Note the shorter observation time in the ‘monophasic’ group (*left lower panel*) and the lower percentage of non-relapsing cases among patients with a long observation period (≥8 years; *right lower panel*)

Fourty of 50 MOG-IgG-positive patients (80 %) had a relapsing disease course. In the remaining 10 cases only a single attack had occurred at last follow-up. The proportion of patients with a monophasic course declined with increasing observation time (Fig. 2, *upper panel*). If only patients with a very long observation period (≥8 years) are considered, 93 % (13/14) had a recurrent course (Fig. 2, *lower panel*). In line with this finding, the median observation time was shorter in the ‘monophasic’ than in the relapsing cases (26 vs. 52.5 months). The proportion of patients with a relapsing disease course did not differ significantly between female (83.8 % [31/37]) and male (69.2 % [9/13]) patients.

Symptoms developed acutely or subacutely in the vast majority of cases; progressive deterioration of symptoms was very rare (at least once in 3/46 or 7 %) and reported only in patients with myelitis.

### Clinical presentation during acute attacks

**Fig. 3:**
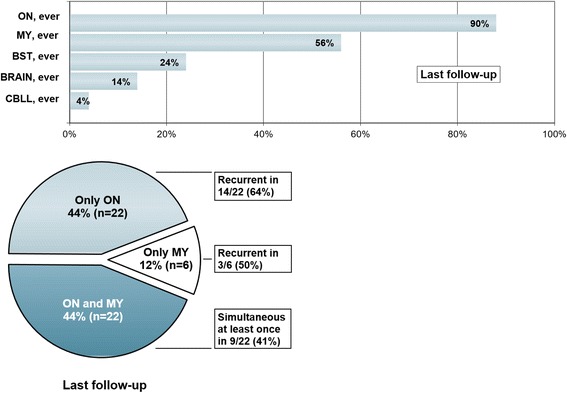
Attack history at last follow-up. *Upper panel:* Frequencies of MOG-IgG-positive patients (*N* = 50) with a history of clinically manifest acute optic neuritis (ON), myelitis (MY), brainstem encephalitis (BST), supratentorial encephalitis (BRAIN), and cerebellitis (CBLL) at last follow-up. *Lower panel:* Frequencies of MOG-IgG patients with a history of optic neuritis (ON) and myelitis, ON but not myelitis, and myelitis (LETM in all cases) but not ON, respectively, at last follow-up (*n* = 50)

Overall, 276 clinically apparent attacks in 50 patients were documented. 205 attacks clinically affected the optic nerve, 73 the spinal cord, 20 the brainstem, 3 the cerebellum, and 9 the supratentorial brain. 44/50 (88 %) patients developed at least once acute ON, 28/50 (56 %) at least once acute myelitis, 12/50 (24 %) at least once a brainstem attack, 2/50 (4 %) acute cerebellitis, and 7/50 (14 %) acute supratentorial encephalitis (Fig. 3, *upper panel*).

At last follow-up, 26/50 (52 %) patients had developed at least two different clinical syndromes (i.e., combinations of ON, myelitis, brainstem encephalitis, cerebellitis, and/or supratentorial encephalitis), either simultaneously or successively. Of these, 22 (84.6 %) had experienced attacks both of ON and of myelitis at last follow-up (corresponding to 44 % [22/50] of the total cohort). Another 22 (44 %) had a history of ON but not of myelitis (recurrent in 15 or 68.2 %), and 6 (12 %) had a history of myelitis but not ON (recurrent in 4; LETM in all) at last follow-up (Fig. 3, *lower panel*).

Myelitis and ON had occurred simultaneously (with and without additional brainstem or brain involvement) at least once in 9/22 (40.9 %) patients with a history of both ON and myelitis at last follow-up (and in 18 % or 9/50 in the total cohort).

Overall, 16/50 (32 %) patients presented at least once with more than one syndrome during a single attack (more than once in 10/16). While 15 attacks of myelitis (without ON) in 11 patients were associated with clinical signs and symptoms of simultaneous brain or brainstem involvement, only 1 attack of ON (without myelitis) in 1 patient had this association. Clinically inapparent spinal cord, brain, or brainstem involvement was detected in further patients by MRI (see Brain MRI findings below and part 3 of this article series [31] for details).

### Symptoms associated with acute myelitis

**Fig. 4:**
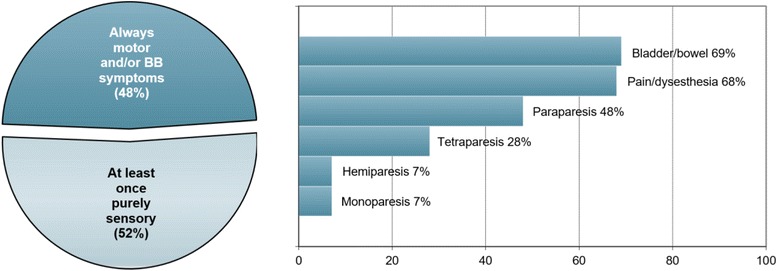
Symptoms present during attacks involving acute myelitis (*N* = 28 patients). BB = bladder and/or bowel.

Symptoms present at least once during attacks of myelitis included tetraparesis in 8/29 (27.6 %) patients, paraparesis in 14/29 (48.3 %), hemiparesis in 2/29 (6.9 %), and monoparesis in 2/29 (6.9 %). Paresis was severe (BMRC grades ≤2) at least once in 6/29 (20.7 %) patients. Attacks included at least once pain and dysesthesia in 19/28 (67.9 %) patients and were purely sensory in 15/29 (51.7 %). Sensory symptoms included also Lhermitte’s sign. Bladder and/or bowel and/or erectile dysfunction occurred at least once in 20/29 (69 %) patients (Fig. 4).

### Symptoms associated with acute ON

In 36/39 (92.3 %) patients ON was associated with reduced high-contrast visual acuity (VA) as determined using a Snellen chart. In one patient, low-contrast but not high-contrast VA was reduced; in another patient with hazy vision but normal high-contrast VA, low-contrast VA was not tested. In a third patient, impaired color perception and papilledema were the only clinical symptoms.

Most patients with ON reported retrobulbar pain and/or pain on eye movement. Disturbed color vision including color desaturation was reported in some patients, but was not systematically examined in all patients.

**Fig. 5:**
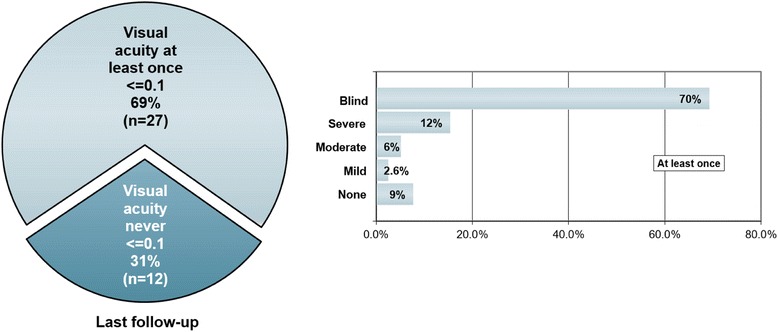
High-contrast visual acuity (VA) loss during acute ON (*N* = 39 patients). Blind: complete or functional blindness (VA ≤0.1) in one or both eyes at least once; severe: VA ≤0.5; moderate: VA ≤0.75; mild: ≤1.0; none: high-contrast VA not affected, but low-contrast visual loss, color desaturation, and/or scotoma present

Attack-related functional blindness (defined as VA ≤0.1) in one or both eyes occurred at least once in 27/39 (69.2 %) patients and VA ≤0.5 was present at least once in 33/39 (84.6 %) during acute ON attacks (Fig. 5). Both eyes were affected simultaneously (‘bilateral ON’) at least once in 22/43 (51.2 %) patients, and scotoma was noted at least once in 23/35 (65.7 %) with available data.

### Other symptoms

Brainstem symptoms occurred in 12 MOG-IgG-positive patients. A detailed analysis can be found in part 3 of this article series [31]. Respiratory insufficiency due to brainstem encephalitis (2 ×) or myelitis (1 ×) occurred at least once in 3/48 (6.3 %) patients with available data (median observation time 50.5 months; range 1-507) and was fatal in one of these two cases. Two patients had clinical signs and symptoms indicating cerebellar involvement. These included limb, gait, and stance ataxia with or without accompanying dysarthria. Sensory ataxia was noted in others.

Supratentorial brain lesions were symptomatic in 7 patients. These patients showed (sometimes severe) headache, fatigue, psychomotor slowing, disorientation, impaired consciousness/somnolence, hemihypesthesia, meningism, and photophobia.

Of note, several further patients had brainstem, cerebellar, and/or supratentorial brain lesions (see section Brain MRI findings below and Appendix as well as part 3 of this article series [31]) but no clinical symptoms attributable to those lesions.

### Presentation at onset

**Fig. 6:**
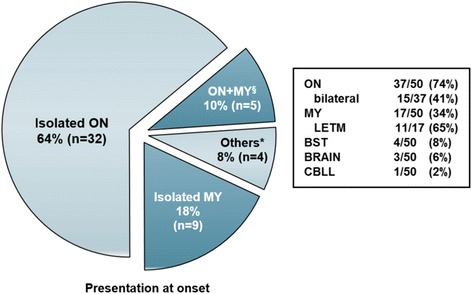
Presentation at onset. ON = optic neuritis, MY = myelitis, LETM = longitudinally extensive transverse myelitis, BST = brainstem encephalitis, BRAIN = supratentorial encephalitis, CBLL = cerebellitis. ^§^ Includes two cases of simultaneous ON, myelitis and brainstem encephalitis at onset. ^*^Other presentations included simultaneous myelitis, rhombencephalitis and supratentorial encephalitis; simultaneous myelitis and supratentorial encephalitis (2 ×); and isolated brainstem encephalitis. No data on spinal cord lesion length at disease onset were available from 1 patient

ON was clearly the most common manifestation at disease onset (present in 37/50 [74 %] patients), followed by myelitis (17/50 [34 %]), brainstem encephalitis (4/50 [8 %]) and symptoms attributable to brain (3/50 [6 %]) or cerebellar lesions (1/50 [2 %]). While in some patients only one site was clinically affected, multiple manifestations were noted in others: thirty-two of 50 patients (64 %) initially presented with isolated ON; 9 (18 %) with isolated myelitis; 5 (10 %) with simultaneous ON and myelitis (additional brainstem involvement in 2); 1 (2 %) with simultaneous myelitis, rhombencephalitis, and supratentorial encephalitis; 2 (4 %) with myelitis and supratentorial encephalitis; and 1 (2 %) with isolated brainstem encephalitis (Fig. 6). Accordingly, clinical evidence for dissemination in space (here understood as involvement of more than one of the following anatomical sites: optic nerve, spinal cord, prosencephalon, brainstem, and/or cerebellum) was present at onset in 8/50 (16 %) patients (compared to 16/50 (32 %) if the entire observation period is considered).

In the subgroup of patients with multiple manifestations at follow-up (including NMO and any other combinations of ON, myelitis, brainstem encephalitis, cerebellitis, and/or supratentorial encephalitis) (*N* = 26), disease had started with an isolated syndrome in 17 (65.4 %) (isolated ON in 12 [46.2 %] and isolated myelitis in 5 [36.4 %]); with simultaneous ON and myelitis in 4 (15.4 %); with simultaneous ON, myelitis, and brainstem encephalitis in 1 (3.8 %); with simultaneous myelitis, rhombencephalitis and supratentorial encephalitis in 1 (3.8 %); and with simultaneous myelitis and supratentorial encephalitis in 2 (7.7 %).

In the subgroup of patients meeting Wingerchuk’s 2006 criteria at last follow-up, 3/14 (21.4 %) had simultaneous ON and myelitis at onset (exclusively or in combination with brain, brainstem or cerebellar symptoms) and 3/8 (37.5 %) of those with ON at disease onset, including 2 of the 3 cases with simultaneous ON and myelitis – presented with bilateral ON.

The initial attack affected both eyes in 15/37 (40.5 %) of all patients with ON at onset and in 11/32 (34.4 %) of all patients with isolated ON at onset; overall, 15/50 (30 %) patients had bilateral ON at onset (partly in combination with other manifestations).

The first attack of myelitis was clinically characterized by tetraparesis in 5 patients and by paraparesis in 6; in 5 patients, myelitis was associated with purely sensory and/or autonomous symptoms at onset. In 2 patients, respiratory dysfunction was among the presenting symptoms.

### Time to second attack

**Fig. 7:**
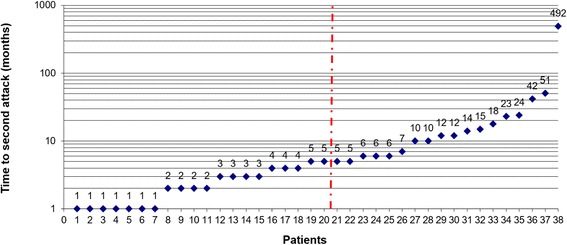
Time to first relapse in months. The *red line* indicates the median. The first relapse was defined as a new clinical attack occurring more than 30 days after onset of the initial attack. No exact data was available in two cases

Among the MOG-IgG-positive patients with more than one documented attack and available data, the median time between the first and the second attack was just 5 months (range, 1-492; N = 38) (Fig. 7). There was no significant difference between patients with ON at onset (median of 6 months to next relapse; range 1-492) and patients with myelitis at onset (median 4 months; range 1-23). The median interval between first and second attack was slightly longer among patients with full recovery from the first attack (*n* = 17) than in the remaining patients (6 vs. 3.5 months; *p* = n.s.).

### Presentation at second attack

The most common manifestation (isolated [*N* = 22] or in combination with other syndromes) at second attack was ON (21/23 [91.3 %], which was mostly unilateral (21/23 [91.3 %]; no data in one case). Other presentations at second attack included isolated myelitis (*N* = 12), isolated supratentorial encephalitis (*N* = 1), myelitis with brain or brainstem involvement (*N* = 2), and simultaneous ON and myelitis with brain involvement.

The initial presentation had high predictive value for the second attack: in 18 of 25 patients (72 %) initially presenting with isolated ON, the second event was isolated ON again (and in 19/25 or 76 % patients, ON was among the presenting manifestations); similarly, in 6/8 (75 %) patients with isolated myelitis the second event was also isolated myelitis. Overall, at least one manifestation present at onset (ON, myelitis, brainstem encephalitis, cerebellitis, supratentorial encephalitis) was present also at the second attack in 31/40 (78 %) patients with a recurrent disease course.

Of note, both optic nerves were affected clinically early in the disease course: in 6/10 (60 %) patients with available data who experienced a unilateral ON at disease onset and ON at first relapse, the second attack affected the previously unaffected eye (or both eyes). Overall, 21/34 (62 %) patients had a history of ON in both optic nerves (simultaneously or subsequently) already after the second event.

### Annualized relapse rate

If all patients with an observation time of ≥12 months are considered, the median annualized relapse rate (ARR) was 0.83 (range 0.05-6.92) in the total group (*n* = 39) and 0.92 (range 0.05-6.92) among patients with a recurrent disease course (*n* = 34). It was higher among female than among male patients both in the total cohort (0.92 vs. 0.535; *N* = 29 and 10, respectively) and in the relapsing subgroup (0.92 vs. 0.83; *N* = 27 and 7, respectively), but the differences were not statistically significant.

The median ARR was highest (1.17; range 0.05-4.2; *N* = 19) in relapsing patients with a history of both ON and myelitis (*n* = 21), compared with 0.8 (range 0.5-6.92) among patients with recurrent isolated ON but no myelitis (*n* = 12) and 0.57 and 0.83 in the two only patients with recurrent isolated LETM but no ON and an observation time ≥12 months.

### Brain MRI findings

**Fig. 8:**
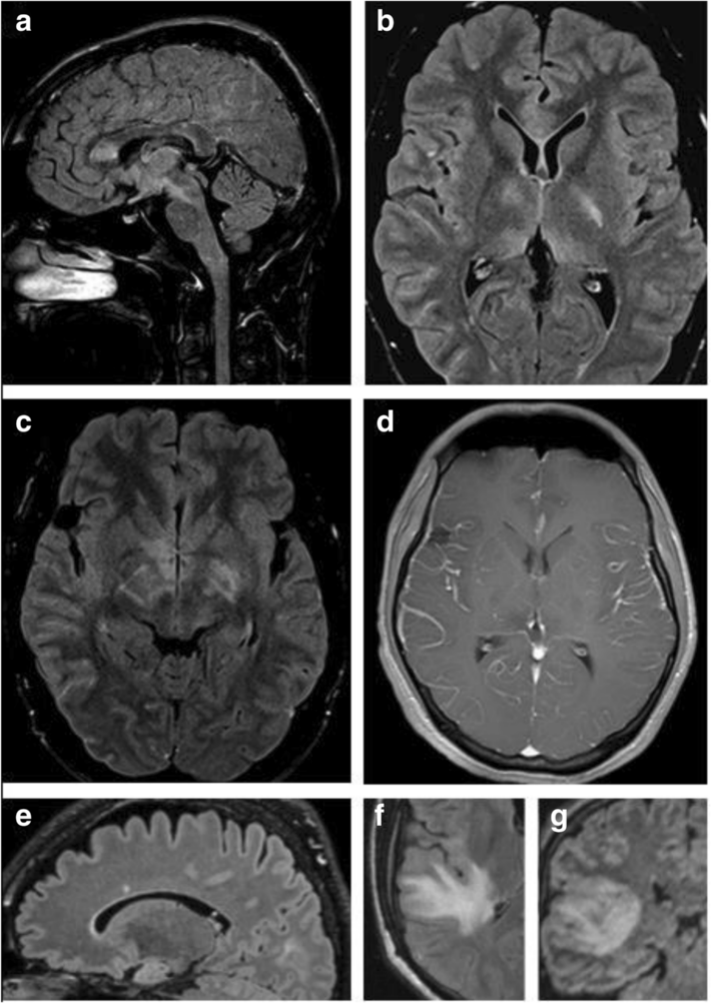
Examples of brain lesions detected by MRI. **a** Sagittal FLAIR image showing callosal lesions as well as lesions extending from the diencephalon to the pons (see case 8 in part 3 of this article series [31] for details). **b** Axial FLAIR MRI demonstrating lesions in the basal ganglia, juxtacortically on the right side, und in the genu corporis callosi in the same patient. **c** Axial FLAIR image at the diencephalic level revealing periependymal lesions (in addition to basal ganglia lesions). **d** Axial T1-weighted image with Gd demonstrating leptomeningeal enhancement (see case 8 in part 3 [31]). E: Sagittal MRI showing a callosal lesion (see case 10 in the Appendix for details). **f**, **g** Axial T2-weighted (**f**) and coronal FLAIR (**g**) images showing large, confluent T2 hyperintense lesions in the right temporal lobe (see case 7 in part 3 [31])

Supratentorial MRI abnormalities were present at onset in 17/48 (35.4 %) MOG-IgG-positive patients and infratentorial MRI lesions in 7/48 (14.6 %). Supratentorial MRI lesions at onset included periventricular lesions; lesions in the corpus callosum (some of them confluent); frontal, parietal, temporoparietal, and occipital deep white matter lesions; subcortical or juxtacortical lesions (including insular lesions); and, in one case, lesions in the thalamus (pulvinar) and in the basal ganglia (putamen) (Fig. 8). In one patient leptomeningeal enhancement was noted at onset (Fig. 8, panel d), and in one both optic tracts were affected (Fig. 8, panel c).

Infratentorial lesions at onset included lesions in the cerebral peduncles, the pons (incluing tegmentum), medulla oblongata, cerebellar hemispheres, and cerebellar peduncles (see part 3 of this series [31] for details).

Taking not only the first but all MRIs into account, 22/47 (46.8 %) patients had supratentorial brain lesions at least once; brainstem lesions occurred at least once in 14/48 (29.2 %); and cerebellar lesions were noted at least once in 6/48 (12.5 %) (see part 3 of this series for details [31]). Lesions affected the periventricular white matter, deep white matter (in some cases large and confluent) and corona radiata, sub- or juxtacortical white matter, corpus callosum, thalamus (pulvinar), basal ganglia, cerebral peduncles, pons (ventral, median, tegmentum), medulla oblongata (including the area postrema and the periaqueductal gray), cerebellar hemispheres, and cerebellar peduncles and were partly Gd-enhancing. Lesions were found in the frontal, parietal, temporal, and occipital lobes and in the insula. Taking the entire course of disease into account, callosal lesions were present at least once in 8/48 (16.7 %) patients and periventricular lesions in 12/47 (25.5 %). Callosal lesions were longitudinally extensive (more than half the length of the corpus callosum), as considered typical for AQP4-IgG-positive NMOSD [29], in 1/8 (12.5 %).

### Optic nerve MRI findings

**Fig. 9:**
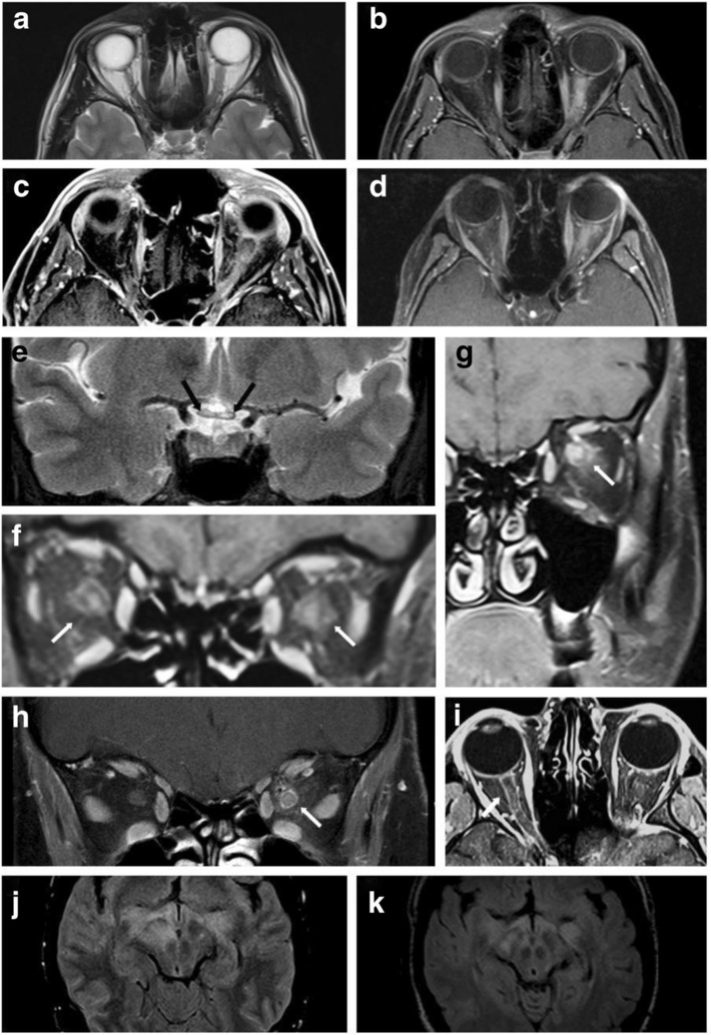
Examples of optic nerve lesions detected by MRI. **a**, **b** T2-weighted (**a**) and T1-weighted (B, with Gd) MRI reveals swelling and Gd enhancement of the left optic nerve. **c**, **d** (fat-suppressed): Longitudinal extensive Gd enhancement of the optic nerve (see cases 9 and 12 in part 3 [31] for details). **e** Longitudinally extensive bilateral optic neuritis extending from the chiasm (E, *black arrows*) into the orbits, affecting the left more than the right optic nerve. **f**-**h** Coronal T1-weighted MRIs display marked contrast enhancement of the intraorbital optic nerve as well as concurrent enhancement of the perioptic nerve sheath, partly extending in the surrounding orbital fat, in patients with acute ON (cases 11, 29 and 19). I: Axial T1-weighted MRI shows Gd enhancement along the right optic nerve in another patient (see case 13 in part 3 of this article series [31]). **j**, **k** Axial FLAIR imaging demonstrates bilateral lesions in the optic tract (see case 8 in part 3 [31] for details) (**j** MRI at attack onset; **k** follow-up MRI 1 month later)

MRI signs of ON were present in at least 24/44 (54.5 %) patients with available data, all of whom had a history of clinical ON (Fig. 9). Intraorbital swelling of the optic nerve was noted at least once in 13/21 (61.9 %) patients, and contrast enhancement in 20/21 (95.2 %). A longitudinally extensive optic nerve lesion (more than half the length of the nerve) (*n* = 6) and/or involvement of the optic chiasm (*n* = 4), two findings previously considered typical for AQP4-IgG-positive NMO [29], were present during acute ON in 8/26 (30.8 %) cases with available data. Signs of optic nerve atrophy were noted in at least 5 patients and involved the optic chiasm in at least one of them. However, post-chiasmatic parts of the optic pathway were also affected in individual patients: as mentioned above, one patient had optic tract lesions, and occipital lobe white matter lesions were documented in four cases.

Of particular note, in 11/28 (39.3 %) patients with available data, perioptic contrast enhancement, i.e. gadolinium enhancement within the nerve sheath and the immediately surrounding orbital tissues, was present during acute ON (Fig. 9). The remaining patients had either no history of ON or no or no suitable post-contrast orbital MRI was performed or retrospectively available for re-analysis and the presence of absence of perioptic enhancement was not mentioned in their MRI reports.

### Spinal cord MRI findings

**Fig. 10:**
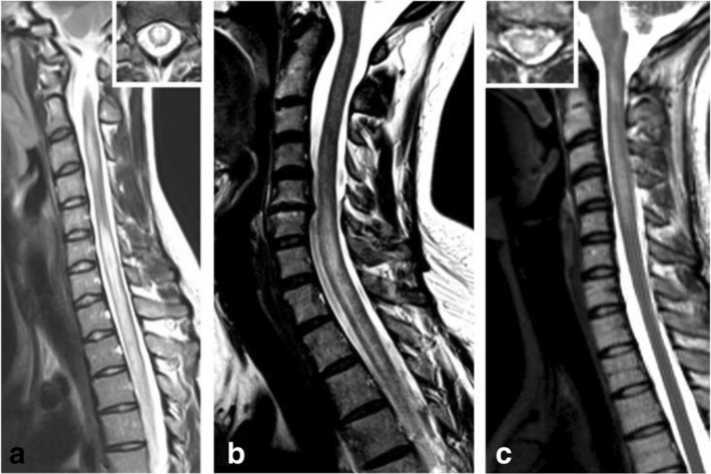
Examples of spinal cord MRI findings. **a** Sagittal T2-weighted spinal MRI performed at disease onset revealed a large longitudinal centrally located lesion extending over the entire spinal cord as well as swelling of the cord. **b** Longitudinal extensive central spinal cord T2 lesion in another patient. **c** T2-hyperintense lesions extending from the pontomedullary junction throughout the cervical cord to C5 in a third patient. The *insets* in A and C show axial sections of the thoracic cord at lesion level

MRI signs of spinal cord inflammation were present in 29/44 (65.9 %) patients with available data, including 27/28 (96.4 %) with a history of clinical myelitis (Fig. 10).

Spinal MRI was performed also in 16 patients without a history of clinically apparent myelitis and showed a spinal cord lesion extending over 2 segments in 2 of them.

In 20 out of the 28 (71.4 %) patients with a history of clinical myelitis and available data, two or more lesions were present simultaneously (i.e., in the same MRI) at least once.

Spinal cord lesions on MRI extending over three or more vertebral segments (VS), i.e., so-called LETM lesions, were documented in 21/29 (72.4 %) patients at least once. LETM lesions were present during the first attack in 11/17 (64.7 %) patients initially presenting with acute myelits.

By contrast, in 8 patients exclusively short lesions (<3 VS), i.e., so-called non-longitudinally extensive transverse myelitis (NETM) lesions, were documented over the entire observation period. Of potential differential diagnostic importance, spinal cord MRI showed one or more NETM lesions but no LETM lesions at disease onset in 6/17 (35.3 %) patients initially presenting with acute myelitis (alone or in combination with other syndromes). If all available MRIs are considered, MRI lesions extended over fewer than three segments during acute attacks of myelitis in 12/27 (44.4 %) patients.

The median length of all documented LETM lesions (*n* = 32) was 4 VS (range 3-20) and that of all documented NETM lesions (*n* = 44) was 1.5 VS (range 1-2). If all spinal cord lesions with available data are considered (*n* = 76), i.e., both LETM and NETM lesions (including NETM lesions present in addition to LETM lesions in the same MRI), the median longitudinal extension was 2 VS (range 1-20). Finally, the median length of the longest spinal cord lesion (LETM or NETM) ever observed in each patient was 5 VS (range, 1-20; *N* = 27) if all patients with available MRI data were considered and 5 (range, 1-20; *N* = 26) if only patients with clinical evidence for myelitis were considered.

Swelling of the spinal cord was noted at least once in 19/27 (70.4 %) patients and contrast enhancement in 19/28 (67.9 %). Signs of necrosis of the spinal cord were noted in 0/23 (0 %) patients with available data.

Spinal cord lesions were located in the cervical spinal cord at least once in 23/28 (82.1 %) patients and in the thoracic spinal cord at least once in 21/28 (75 %). Lumbar and conus lesions were documented only in 3/27 (11.1 %) and 3/27 (11.1 %) patients, respectively. Taking all available spinal cord MRIs into account, cervical lesions were present in 44/81 (54.3 %) MRIs, thoracic lesions in 31/81 (38.3 %), lumbar lesions in 4/81 (4.9 %), and the conus was affected in 3/81 (3.7 %). However, as a limitation, not all MRIs performed showed the entire spinal cord, and spinal cord MRI data were absent for 18 myelitis attacks in 8 patients.

Information on intramedullary lesion location was available for 34 lesions in 20 MOG-IgG-positive patients. Lesions were located predominantly in the central portion of the spinal cord in 17 MRIs and predominantly in the peripheral portion in another 17 MRIs.

The spinal cord MRI was normal during 2 attacks; in both cases, symptoms were purely sensory (paresthesia and hyp- and dysesthesia, respectively). Of note, a total of five asymptomatic spinal cord lesions were noted in two patients (in addition to brainstem lesions in one) with a history of ON but no clinical evidence of myelitis over the course of disease.

### Evaluation of Barkhof’s and Paty’s MRI criteria for MS

Seven of 46 (15.2 %) MOG-IgG-positive patients with a history of myelitis and/or ON and 7/26 (26.9 %) of those with brain lesions met Barkhof’s MRI criteria for MS at least once [38]. However, at least 2 of the 7 patients meeting Barkhof’s criteria also had one or more NMOSD-typical lesions at least once.

The revised 2006 diagnostic criteria for NMO [28] required a brain MRI at disease onset that does not meet Paty’s MRI criteria for MS [39] if either no LETM lesion is present or NMO-IgG is negative. Accordingly, Paty’s criteria were evaluated only at disease onset. In the present cohort, the initial MRI of 12 out of 48 (25 %) MOG-IgG-positive patients with available data met Paty’s criteria.

### Intrathecal IgG synthesis

Data on CSF-restricted oligoclonal IgG bands (OCB) were available from 45/50 (90 %) MOG-IgG-positive patients. Pattern 2 or 3 OCB [40] indicative of intrathecal IgG synthesis were positive at least once only in 6/45 (13.3 %). A second lumbar puncture was performed in 2 out of the 6 OCB-positive patients, in both of whom OCB remained positive.

Patients with classical MS display a polyspecific, intrathecal humoral immune response to neurotropic viruses such as measles, rubella, and varicella zoster virus (the so-called MRZ reaction, MRZR) [41–44]. MRZR was tested in 11 MOG-IgG-positive patients (2 x ON + myelitis; 1 x ON + myelitis + brainstem encephalitis; 1 x myelitis + brainstem encephalitis; 3 x LETM; 5 x ON) and was negative in all of them.

### CSF white cell counts

White cell counts (WCC) in the CSF were documented at least once in 46 MOG-IgG-positive patients and were elevated (>5/μl) in 32 (69.6 %). In those patients with pleocytosis, WCC ranged between 6 and 306 cells/μl (median 33; quartile range 13-125). WCC ≥100 cell/μl were present at least once in 9/32 (28.1 %) patients. Neutrophil granulocytes were present at least once in 9/14 (64.3 %) patients with pleocytosis and available data (median 22 % of all white cells; range 3-69 %).

### Blood-CSF barrier function

An increased albumin CSF/serum ratio (QAlb) reflects a disturbed blood-CSF barrier (BCSFB) function caused by structural damage and/or a reduced CSF flow rate [45]. QAlb was determined in 37 MOG-IgG-positive patients and was elevated in 12 (32.4 %). Blood-CSF barrier dysfunction was present both among patients with a history of isolated ON (2/15; 13.3 %) and, more frequently, in patients with a history of spinal cord and/or brain/brainstem involvement (10/21; 47.6 %).

### Visual evoked potentials

Data on visual evoked potentials (VEP) were available from 47 MOG-IgG-positive patients. A delayed P100 latency was noted at least once in 34 (72.3 %); in another 6 (12.8 %) patients latencies could not be determined since potentials were lost due to severe optic nerve damage.

Only 41 (78.7 %) of the 47 patients examined had a history of clinically manifest ON; in 31 of these 41 patients (75.6 %) P100 latency was delayed, and in 6 further patients (14.6 %) latencies could not be determined. The remaining 6 patients had a history of myelitis (LETM in all cases) but no history of clinically manifest ON. 3 of those 6 had delayed P100 latencies in at least one eye, indicating that subclinical optic nerve damage might be relatively frequent in MOG-IgG-positive patients with myelitis.

In 23/41 (56.1 %) patients, all of whom had a history of clinical ON, VEP amplitudes were reduced (*n* = 16) or lost (*n* = 7) at least once. In all but one patient with reduced amplitudes, P100 latencies were also delayed at some point in time, but not vice versa.

### Somatosensory evoked potentials

Data on somatosensory evoked potentials (SSEP) were available from 39 MOG-IgG-positive patients, including 24 with a history of clinically manifest myelitis. SSEP were delayed, reduced in amplitude, or lost in 19/39 (46.2 %), including in 16/24 (66.7 %) with a history of clinical myelitis and available data. Of note, 3 patients with no clinical history of myelitis had SSEP abnormalities suggestive of subclinical spinal cord damage (none of them displayed unequivocal spinal cord MRI abnormalities).

### Ophthalmoscopic findings

Fundoscopy revealed uni- or bilateral papillitis or papilledema in at least 15 patients with acute ON, suggesting inflammation of the anterior part of the optic nerve. The true prevalence of papillitis could be higher, however, since ophthalmoscopic data were not available from all patients. In case 6 (see Appendix), papilledema was described as marked (3 dpt) at first ON and as mild at second and third ON, while later on the optic disk was described as atrophic and pale. Optic atrophy as detected by fundoscopy was noted at last follow-up in 13/22 (59.1 %) patients with available data.

### Evaluation of the 2010 McDonald criteria for MS

If MOG-IgG seropositivity is not considered to constitute per se a “better explanation” [46], i.e., based solely on clinicoradiological criteria, 15/46 or 33 % of the patients with available data met the most current diagnostic criteria for MS [46] (Table 1). Taking only MOG-IgG-positive patients with a history of both ON and myelitis into account, 10/20 or 50 % with available data fulfilled those criteria, compared with 7/31 or 23 % with a history of ON but not myelitis or of myelitis but not ON at last follow-up. If only patients with a relapsing disease course are taken into account, 44 % (15/34) met the 2010 McDonald criteria.

**Table 1:** Patient numbers and diagnoses

Diagnostic categories	*N* (%)
History of ON and/or MY	50/50 (100 %)
History of ON	44/50 (88 %)
History of myelitis	28/50 (56 %)
Meeting Wingerchuk’s 2006 criteria for NMO^a^	14/50 (28 %)
Meeting 2015 consensus criteria for NMOSD^b^	16/50 (32 %)
Meeting 2010 McDonald criteria for MS^c^	15/46 (33 %)
History of ON and of myelitis	22/50 (44 %)
Meeting Wingerchuk’s 2006 criteria for NMO^a^	14/22 (63.6 %)
Meeting 2015 consensus criteria for NMOSD^b^	15/22 (68.2 %)
History of ON but not of myelitis	22 (44 %)
Meeting Wingerchuk’s 2006 criteria for NMO^a^	0/22 (0 %)
Meeting 2015 consensus criteria for NMOSD^b^	1/22 (4.5 %)
History of myelitis but not of ON	6/50 (12 %)
Meeting Wingerchuk’s 2006 criteria for NMO^a^	0/6 (0 %)
Meeting 2015 consensus criteria for NMOSD^b^	0/6 (0 %)

*MS* multiple sclerosis, *NMO* neuromyelitis optica, *NMOSD* NMO spectrum disorder, *ON* optic neuritis. ^a^see ref. [28], ^b^see ref. [29], ^c^see ref. [46]

### Evaluation of the 2006 criteria for NMO

63.6 % (14/22) of all MOG-IgG-positive patients with a history of both ON and myelitis met Wingerchuk’s 2006 revised diagnostic criteria for NMO [28] (Table 1). Of the 8 patients with ON and myelitis who did not meet Wingerchuk’s 2006 criteria, two had an LETM lesion but the first brain MRI met Paty’s criteria for MS; five did not meet Paty’s criteria at onset but spinal cord lesions extended over fewer than three vertebral segments; and one met Paty’s criteria at onset and had no LETM lesion.

Twenty eight patients had a history of ON but not myelitis or a history of myelitis but not ON (both with and without brain involvement) and did therefore not meet the 2006 diagnostic criteria. Taking the total cohort into account, 28 % (14/50) of all patients met the 2006 criteria for NMO. Seven out of 43 (16 %) patients with available data fulfilled both the clinicoradiological 2006 criteria for NMO [28] and the clinicoradiological 2010 McDonald criteria for MS [46].

### Evaluation of the 2015 criteria for NMOSD

On the understanding that MOG-IgG seropositivity does not per se constitute an “alternative diagnosis”, i.e., based solely on clinical and radiological criteria, 16/50 (32 %) patients met the 2015 international consensus criteria for NMOSD [29] (Table 1). Of those, 15 had a history of both ON and myelitis and 1 a history of ON but not of myelitis (this patient fulfilled the criteria despite the lack of myelitis due to the presence of brainstem encephalitis with periependymal lesions around the fourth ventricle and of symptomatic, extensive white matter lesions); none had a history of myelitis but not of ON. Of those patients who met the 2006 criteria, 12 (85.7 %) also met the 2015 criteria. Conversely, 12 (75 %) of those who met the 2015 criteria also met the 2006 criteria. 8 out of 43 (19 %) patients with available data fulfilled both the clinicoradiological 2015 criteria for NMOSD and the clinicoradiological 2010 McDonald criteria for MS. If only patients with a relapsing course of disease are considered, 16/40 (40 %) met Wingerchuk’s 2015 criteria.

### Previous diagnoses

As reliable tests for MOG-IgG became available only relatively recently, most of the patients initially received diagnoses other than MOG-IgG-positive encephalomyelitis (EM). In 16/45 (35.6 %) patients with available data, a diagnosis of MS was suspected at least once. Other suspected diagnoses included acute disseminated EM (ADEM), multiphasic disseminated EM, AQP4-IgG-negative NMO according to Wingerchuk’s 2006 criteria [28], AQP4-IgG-negative NMOSD according to the 2015 international diagnostic consensus criteria [29], viral encephalitis, bacterial encephalitis, paraneoplastic encephalitis, isolated vasculitis of the CNS, chronic relapsing inflammatory optic neuropathy (CRION), CNS lymphoma, sarcoidosis, spinal stenosis, “spinal tumor of unknown dignity”, suspected spinal ischemia, para- or postinfectious ON, and myelitis; some patients were diagnosed with ON, rON, (longitudinally extensive transverse) myelitis, brainstem encephalitis or EM “of unknown origin”.

### Coexisting autoimmunity

Coexisting autoantibodies were present in 19/45 (42.2 %) MOG-IgG-positive patients. These included antinuclear antibodies (ANA) in at least 14 patients and cardiolipin antibodies or phospholipid/glycoprotein beta-2 antibodies (2 ×), anti-tissue transglutaminase IgA (1 ×), rheumatoid factor (1 ×), anti-thyroid peroxidase (2 ×), anti-thyreoglobulin (1 ×), anti-thyroid-simulating hormone receptor (1 ×), perinuclear anti-neutrophil cytoplasmic antibodies (ANCA) (1×). None of the 50 patients was positive for AQP4-IgG [30].

Concomitant autoimmune disorders were present only in 4/47 (8.5 %) patients and included rheumatoid arthritis (RA) (2 ×), Hashimoto thyroiditis (1 ×), Grave’s disease (1 ×). A further patients had atopic dermatitis and asthma bronchiale.

### Preceding infections

Disease onset was preceded by infection in at least 11 patients. Diagnoses included common cold, sore throat, tonsillitis, sinusitis, bronchitis, “respiratory infection”, “feverish infection”, and, in one case, a gastrointestinal infection with positive *Yersinia* serology (species not determined).

Taking not only the first but all attacks into account, attacks were preceded by infection at least once in at least 15/37 (40.5 %) patients; the infections included, in addition to those already mentioned above, “mycoplasma pneumonia,” one case each of a non-specified “respiratory” or “bronchopulmonary” infection, a “feverish common cold”, “fever and fatigue”, and a non-specified “feverish infection”. In at least one patient, both the first and the second attack were preceded by infection.

One further patient reported a history of two episodes of “borreliosis with meningitis” 20 and 19 years before onset.

### Preceding vaccinations

Disease onset was preceded by revaccination against diphtheria, tetanus, pertussis, polio, and influenza 2 weeks prior to symptom onset in one patient (for details of this case see part 3 of this series [31]), and by vaccination against diphtheria, tetanus, and pertussis 13 days prior to symptom onset in a second case; the latter patient developed fever 2 - 3 days before symptoms started. Both patients (1 × male, 1 × female) were vaccinated at adult age (19 and 47 years) and both developed recurrent disease. While the first patient experienced seven relapses involving the the optic nerves (4 ×), spinal cord (5 ×), brain (2 ×), and brainstem (1 ×) within 20 months, which fully responded to IVMP or combined IVMP and plasma exchange (PEX), the second patient developed three attacks (2 × ON, 1 × myelitis and ON) within 6 months, which only partially responded to IVMP, PEX and IA and resulted in an EDSS of 8 at discharge; two of the attacks occurred despite treatment with rituximab.

### Pregnancy-associated attacks

Seventeen percent (5/30) of all female patients aged ≥15 years at last follow-up experienced at least one attack of ON or myelitis during pregnancy or post partum. This corresponded to 50 % (5/10) of all patients with a documented pregnancy (no data in 9) and, importantly, included all 5/5 women of reproductive age with available data who were pregnant shortly before (i.e., within the last 18 months), at, or after disease onset. Of a total of seven attacks, three had occurred during pregnancy and four post partum. These included the first attack ever in 3 patients: Disease started with simultaneous ON and LETM and accompanying brainstem and brain lesions occurring just 6 weeks after the delivery of the first child in one case; with an attack of unilateral ON 3 months post partum and during breast-feeding in a second patient; and with an attack of bilateral ON 8 months after delivery and while still breast-feeding in a third (as a limitation, however, ON was also preceded by a common cold with mild fever in this last case). In a fourth patient, an attack of LETM occurred during week 6 of pregnancy and an attack of bilateral ON a few weeks after delivery; however, the disease had started 8 years earlier in this patient and several ON attacks had occurred in the meanwhile. A fifth patient experienced at least two attacks of ON during pregnancy, which responded well to IVMP; disease had started 2 years before. While 3 patients had a relapsing course, 2 have not developed further attacks so far, although the follow-up time is short (6 and 3 months, respectively). Overall, 7/23 attacks in the 3 relapsing patients were associated with pregnancy or delivery, while the majority of attacks were not.

### Tumor associations

In a single patient presenting with post-infectious whole-spine myelitis and severe brainstem and brain inflammation, a mature cystic ovarian teratoma had been removed 2 months before onset of the neurological symptoms, but no signs of malignancy had been found; NMDAR antibodies were negative. In the same patient, a ganglioneuroma was found and resected at a later date. MOG-IgG were not associated with malign tumors also in all other patients studied.

### Treatments for acute attacks

Acute attacks were treated with high-dose IVMP at least once in 47/48 (97.9 %) MOG-IgG-positive patients, with PEX at least once in 19/48 (39.6 %), and with immunoadsorption (IA) in two. Other treatments included oral steroids or dexamethasone i.v. followed by oral steroids in single patients as well as acyclovir and/or antibiotics for pragmatic treatment of initially suspected CNS infection.

Overall, 136 documented attacks were treated with IVMP, 15 with PEX, and 25 with both IVMP and PEX or – in five of them - IA; 18 were not treated at all. PEX or IA were used to treat 20 ON attacks, 16 myelitis attacks (with or without brain and/or brainstem and/or cerebellum involvement), 3 attack of simultaneous ON and myelitis (with or without additional clinical brain involvement), and 1 pure brainstem attack.

### Overall outcome of acute attacks

**Fig. 11:**
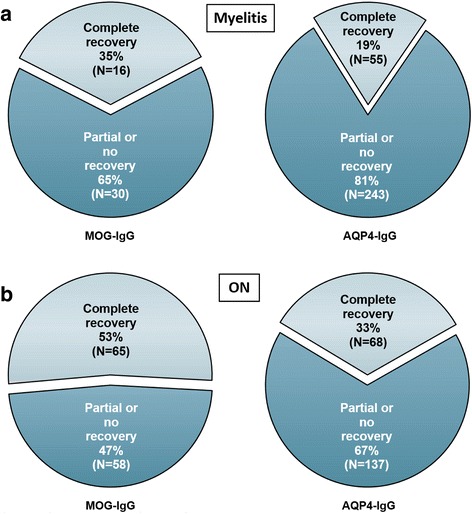
Outcome after acute attacks in MOG-IgG-positive patients compared with a previously published AQP4-IgG-positive cohort. **a** Outcome after acute myelitis in MOG-IgG-positive (46 evaluable attacks) and in AQP4-IgG-positive patients (298 evaluable attacks [34]). **b** Outcome after acute ON in MOG-IgG-positive (134 evaluable attacks) and in AQP4-IgG-positive patients (205 evaluable attacks; see ref. [34]). Note that ‘complete recovery’ includes ‘almost complete recovery’ in the *left graph* (no such distinction was made in the AQP4-IgG-positive cohort)

Outcome data were available for 134 ON attacks in 39 MOG-IgG-positive patients and for 46 myelitis attacks in 23 MOG-IgG-positive patients. Complete or almost complete recovery from acute ON was noted after 70 (52.2 %) ON attacks, partial recovery after 54 (40.3 %), and no or almost no recovery after 10 (Fig. 11b). Complete or almost complete recovery from acute myelitis was noted in 16 (34.8 %) attacks, partial recovery in 30 (65.2 %), and no or almost no recovery in none (Fig. 11a).

At last follow-up, 38/48 (79.2 %) patients had experienced complete or almost complete recovery from at least one attack. In contrast, 22/48 (45.8 %) had experienced at least one attack that was followed by no or almost no recovery. While 62.2 % (28/45) of the patients’ initial attacks remitted completely or almost completely, the proportion was lower for all subsequent attacks (40.6 % or 69/170) and dropped to 26.4 % or 19/72 after the fifth relapse.

### Outcome of attacks treated with IVMP

Outcome data were available for 122 attacks treated with IVMP but not PEX (including attacks of ON; myelitis; brainstem encephalitis; cerebellitis; supratentorial encephalitis; simultaneous ON and myelitis; simultaneous ON, myelitis, and brainstem encephalitis; simultaneous ON, myelitis, and supratentorial encephalitis; simultaneous myelitis and brainstem encephalitis; simultaneous myelitis and supratentorial encephalitis; and simultaneous myelitis, brainstem, and brain inflammation). In 61 (50 %) of those relapses, IVMP treatment was followed by complete or almost complete recovery, in 54 (44.3 %) by partial recovery, and in 7 (5.7 %) by no or almost no recovery.

Of particular note, symptoms flared up after withdrawal or tapering of steroids at least once in 21/47 (44.7 %) patients (see Appendix and Discussion for details). To control symptoms, IVMP was combined with or escalated to PEX or IA in 17/48 (35.4 %) patients at least once and for 9.1 % (25/276) of all documented attacks. If those attacks that were subsequently treated with PEX are also taken into account and on the understanding that the use of PEX after IVMP implies partial or full IVMP failure, 86/147 (58.5 %) attacks initially treated with IVMP responded only partially or not at all to IVMP, while IVMP was followed by complete or almost complete recovery in 41.5 %.

### Outcome of attacks treated with PEX or IA

Outcome data were available for 40 attacks treated either with PEX/IA alone or with both IVMP and PEX/IA; IA instead of PEX was used to treat five of those attacks.

Stand-alone PEX/IA was used for treating attacks (*N* = 15) of ON and/or myelitis with and without brain or brainstem involvement and attacks of isolated brainstem encephalitis. The median number of PEX/IA cycles used per attack was 5 (range, 3-11). In 3 (20 %) of those 15 attacks, PEX treatment was followed by complete or almost complete recovery, in 11 (73.3 %) by partial recovery, and in 1 by no or almost no recovery.

In addition, 25 attacks of ON and/or myelitis (with and without brain and/or brainstem involvement) were treated with both IVMP and, subsequently, PEX/IA. In 10 (40 %) of these attacks, PEX/IA treatment was followed by complete or almost complete recovery, in 14 (56 %) by partial recovery, and in 1 by no or almost no recovery.

If all attacks treated with PEX/IA (with or without IVMP) are considered, PEX/IA treatment was followed by complete or almost complete recovery in 13 (32.5 %) attacks, by partial recovery in 25 (62.5 %), and in 2 (5 %) by no or almost no recovery.

IA was used instead of PEX for two attacks of ON in case 11. While treatment with four courses of IA was followed by almost complete recovery from an ON attack that had responded only transiently to a first IVMP cycle (and not at all to a second one) and by a relapse-free period of 3 years, the next ON attack responded only partially to IVMP and four courses of IA. The reason for the differential response to IA during those two relapses is unknown, but, as with PEX, differences in antibody titers as well as timing issues might have played a role. In case 28, IA resulted only in partial recovery when used after IVMP to treat three attacks of isolated myelitis, simultaneous ON and myelitis, and of isolated ON, respectively.

### Outcome of untreated attacks

Only 14 attacks in 11 patients were not treated with steroids or PEX/IA. Among those attacks, no or almost no recovery was noted in 2 cases (acute ON and brainstem encephalitis in one patient, ON in a second), one of which was fatal, partial recovery in 3 (acute ON in all), and full or almost full recovery in 9 (acute ON in 7, acute encephalitis/brainstem encephalitis in 2 ). The reasons for not treating patients for acute attacks were not specified in all cases. IVMP treatment was declined by at least two patients once each (no recovery in one and full recovery in the other one), and a decision in favor of palliative care had been previously made in another patient; in at least one case, ON was considered mild and therefore left untreated.

### Long-term treatments

Long-term immunosuppressive (IS) or immunomodulatory (IM) treatments were used at least once in 35/49 (71.4 %) patients and included azathioprine (AZA) in 18, methotrexate (MTX) in 8, rituximab in 16, glatiramer acetate (GLAT) in 5, interferon-beta (IFN-beta) in 4, natalizumab (NAT) in 3, ofatumumab in 1, intravenous immunoglobulins (IVIG) in 1, mitoxantrone in 2, ciclosporin in 1, mycophenolate mofetil in 1, and oral steroids in 5; 14 patients (including 8 with a so far monophasic disease course) never received any IS/IM treatment.

Breakthrough attacks were noted in 21/31 (67.7 %) patients treated with IS/IM at least once.

### Response to AZA treatment

Data on acute attacks during AZA therapy were available from 17/18 patients treated. The median treatment period was 10 months (range 2-101). Of these 17 patients, 14 (82.4 %) experienced at least one attack under treatment with AZA. In total, 34 attacks occurred under AZA over a cumulative treatment period of 412 months (cumulative ARR 0.99) with a median of 1 attack/patient (range 0-6) in the total AZA group and of 1.5 attacks/patients (range 1-6) in those who had breakthrough relapses.

Of particular note, 14 of the 34 attacks (41 %) took place during the first 6 months, i.e., during the drug-specific latency period. Of these, 11 attacks developed during the first 3 months and only 3 during months 4-6. If all patients are taken into account, the median of all individual ARRs was 2 during the 6-month AZA latency period and 0.92 after the latency period.

Cotreatment with oral steroids or, in a single case, regular PEX was administered only in 9/23 (39.1 %) patients (no data in 2), either for 3 or for 6 months or for the entire treatment period. Importantly, most attacks (12/14) observed during the AZA latency period occurred in patients who were not cotreated. Relapses occurred in only 1 of 14 cotreated patients during the latency period, but in 6/9 patients who were not cotreated. Similarly, 4/5 patients who developed relapses after the latency period were not cotreated, and 14/17 attacks occurring during that period affected non-cotreated patients. Taking the total treatment period into account, 10/12 patients with relapses under AZA were not cotreated at the time of the attack and 26/31 attacks occurred in non-cotreated patients.

### Response to MTX treatment

Data on acute attacks before and during MTX therapy were available from six patients. In case 13 (see Appendix for case reports), a single (though severe and non-remitting) relapse occurred under MTX within 134 months compared with 3 attacks in an 11-month period including 9 months of combined treatment with AZA and oral steroids. As a possible limitation, it remains unclear whether further attacks in the affected right eye went unrecognized due to the pre-existing severe visual deficit. Patient 3 experienced two attacks (both with complete recovery) within a period of 5.5 years of MTX treatment. Of note, however, this included the patient’s first attack ever, which occurred under active MTX treatment for pre-existing RA. MTX was used as treatment for RA also in patient 6 described in part 3 of this article series [31]; in that patient, temporary discontinuation of MTX after 5 years due to severe infection was followed by the first relapse for 40 years. MTX was continued and no further attack occurred over the following 12 months. Similarly, patient 12 in part 3 of this series [31] suffered no attacks during 21 months of MTX treatment, although, three attacks had occurred within 7 months prior to commencement of MTX. Finally, combined treatment with MTX and oral steroids (plus ciclosporin A during the initial 7 months) resulted in disease stabilization in case 6, with only two relapses (with only partial recovery though) in almost 7 years; by contrast, 14 attacks had occurred in the preceding 5 years in this patient (including during treatment with IFN-beta, GLAT, AZA, or rituximab).

Overall, 5 attacks took place in 22.5 years in these patients under treatment with MTX. This corresponds to a cumulative ARR of 0.22, which is lower than the cumulative ARR of 0.95 found among all patients (*n* = 34) with a relapsing disease course. Patient 1, in whom three breakthrough attacks occurred within 8 months of MTX therapy, was the only patient with apparent MTX failure.

### Response to IFN-beta treatment

No decrease in relapse rate was observed under treatment with various IFN-beta preparations, which were given for suspected MS. In case 6, commencement of therapy with i.m. IFN-beta-1a (Avonex®) was followed by two ON relapses 1 and 4 months later. Similarly, s.c. IFN-beta-1a (Rebif®) was followed by an ON relapse less than 2 months after treatment was started. Finally, treatment with s.c. IFN-beta-1b (Betaferon®) was associated with another ON relapse after 2 months. Overall, four relapses occurred within around 16 months of IFN-beta treatment (ARR 3.0). This is in strong contrast to just two ON relapses within 71 months under therapy with MTX and oral steroids in that patient (ARR 0.33). Of interest, both IFN-beta-1a and -1b led to leukopenia. In another patient (see case 5 in part 3 of this article series [31] for details), further relapses occurred and marked disease exacerbation on MRI was noted after the initiation of i.m. IFN-beta-1a treatment, with new spinal and brainstem lesions. In a third patient (case 12), two relapses occurred within 11 months and led to discontinuation of s.c. IFN-beta-1a therapy. A fourth patient experienced an attack of mild ON and myelitis after 8 months of IFN-beta (Rebif®) therapy. When the same patient was again treated with IFN-beta 5 years later (now with Avonex®), an attack of severe unilateral ON occurred two months after treatment initiation and an attack of ON in the opposite eye with simultaneous myelitis after a further 2 months. In total, she experienced three attacks during a total IFN-beta treatment period of 19 months.

### Response to GLAT treatment

Five patients were treated with GLAT for suspected MS. In case 6, no relapse occurred over a period of 6 months; by contrast, four relapses had occurred under IFN-beta over a period of 16 months in the same patient. However, considering the GLAT-specific latency period of 3-6 months observed in MS it remains uncertain whether that decline in relapse rate was due to GLAT treatment or to discontinuation of (potentially disease-exacerbating) IFN-beta treatment. GLAT treatment had to be stopped due to leukopenia in that patient. In case 8, no relapses occurred over a period of 36 months on therapy with GLAT and remission of spinal cord lesions was detected by MRI. However, this patient had previously experienced a relapse-free interval of more than 5 years, rendering it uncertain also in this case whether GLAT was effective. A third patient (see case 1 in part 3 of this article series [31] for details) was relapse-free for almost a year under GLAT, but experienced two relapses (1 x ON, 1 x myelitis) 11 and 13 months after initiation of therapy, leading to discontinuation of GLAT. Previously, one to two relapses per year had occurred over a period of around 6 years, and three relapses within the last 10 months prior to GLAT. A fourth patient (case 14 in part 3 [31]) experienced three ON attacks during 8 months of GLAT treatment; moreover, a further relapse of severe ON leading to transient unilateral blindness occurred a few weeks after GLAT therapy was discontinued. In a further patient (case 13 in part 3 [31]), two attacks occurred during 7 months of treatment with GLAT (3 and 7 months after the first injection). When treated a second time with GLAT more than 3 years later, she experienced a protracted attack of myelitis with paresis, impaired coordination, and impaired ambulation 1 months after commencement of therapy (and thus during the drug’s latency period), which lasted over 2 months and required a total of three cycles of high-dose IVMP therapy.

### Response to NAT treatment

Three patients were treated with NAT for suspected MS. In one of them (see case 1 in [31]), two infusions of NAT were followed by three relapses by 2, 3 and 5 months, which only partially responded to PEX. Treatment with NAT was not continued after the second infusion due to recurrent headache. In the second patient (see case 5 in part 3 [31]) an attack of brainstem encephalitis occurred and MRI showed a new LETM lesion 9 months after commencement of NAT therapy. The third patient (case 13 in part 3 [31]) experienced two myelitis attacks 1 and 4 months after initiation of NAT treatment, followed by a relapse-free interval of 21 months. When NAT was re-initiated 11 months later, she developed two further attacks of myelitis after 4 and 5 months, followed by a relapse-free interval of 9 months; treatment was discontinued due to John Cunningham virus (JCV) seroconversion. In total, four attacks occurred during 29 months of NAT treatment.

### Response to rituximab and ofatumumab

Of 16 patients treated with rituximab at least once, observation periods under rituximab therapy were sufficiently long to allow meaningful analyses of the drug’s efficacy only in 9 patients.

Treatment with rituximab was followed by a decline in relapse rate in 3/9: In one patient (see case 18 in the Appendix), no relapse occurred in 12 months under rituximab compared with four relapses of ON within 6 months beforehand. In another patient (see case 7 in part 3 of this series [31]) one minor relapse with spontaneous remission took place in 28 months, compared with three attacks within the previous 4 months). Finally, in case 12, no relapses occurred during 8 months of rituximab treatment compared with three relapses in the preceding 14 months (two of which, however, took place under treatment with IFN-beta, which was reported to cause disease exacerbation in NMO and which was associated with ongoing or increasing disease activity also in our patients).

Of note, in the other six patients one or more attacks were noted during therapy with rituximab, most of which occurred shortly after rituximab infusion. This is reminiscent of early attacks observed in AQP4-IgG-positive NMO patients treated with rituximab. Two relapses of ON occurred 3 and 7 weeks after the first rituximab infusion (2 × 1000 mg i.v., days 1 and 15) in case 6 (see Appendix). Similarly, patient 1 in part 3 of this series [31] developed severe clinical and radiological deterioration 4 weeks after the first and 2 weeks after the second infusion of rituximab. The latter patient had been treated with PEX 1 month before rituximab was started, indicating that even pretreatment with PEX may not be sufficient in all cases to prevent the risk of rituximab-related attacks. A further patient developed two relapses of ON one months after the first and 2 months after the second infusion, respectively. The fourth patient (case 11 in part 3 [31]) developed severe bilateral ON three months after the second infusion (i.e. four months after the first infusion) of rituximab. A fifth patient developed two attacks of myelitis and of ON 2 months after the first and three months after the second infusion. Finally, one patient who was treated with rituximab for a first attack of myelitis, developed ON just five months after the first infusion of 1000 mg rituximab. By contrast, no early relapses were noted in ten cases.

Of note, two end-of-dose relapses in rituximab-treated patients were documented. One patient (see case 7 in part 3 [31]) relapsed immediately after reappearance of B cells 9 months after the first infusion. Similarly, a relapse occurred in case 6 12 months after the first rituximab infusion. By contrast, CD19 cells were still undetectable and no new relapse has occurred 14 months after onset in case 12.

In one patient (case 13 in part 3 [31]), therapy with rituximab had to be discontinued due to an allergic exanthema.

A single patient was treated with ofatumumab (18 months, four cycles to date). While eight attacks of ON and three attacks of myelitis (one with accompanying brainstem encephalitis) had taken place over a period of 63 months under various previous therapies (ARR 2.1), only a single attack of ON occurred during 18 months (ARR 0.66) of ofatumumab treatment in this patient.

### Response to mitoxantrone and other rare therapies

In the only patient with available data (case 1 in part 3 of this article series [31]), three infusions of mitoxantrone (1 × 12 mg/m^2^ and 2 × 8 mg/m^2^) did not prevent three relapses of myelitis and two of ON within around 5 months, with some of the relapses occurring just a few weeks after infusion. A further patient (case 13 in part 3 [31]) experienced a relapse of sensory myelitis 1 month after initiation of fingolimod. Discontinuation of fingolimod after 3 months due to lymphopenia was immediately followed by a relapse of myelitis with impaired ambulation, paresthesia and dysesthesia below T5, and two flare-ups over the next 2 months, requiring a total of three cycles of (escalating) high dose IVMP therapy.

Ciclosporin was used in combination with MTX and oral steroids in a single patient (see case 6 in the Appendix) for a period of 6 months; no relapses occurred under this regimen. One patient (see case 13 in part 3 [31]) was treated for 4 months with dimethylfumarate. While no relapses occurred during that period, treatment had to be discontinued due to reflux, pharyngitis and laryngitis.

Another patient (case 2 in part 3 [31]) was treated with IVIG over 11 months (and tapering of oral steroids during the initial 3 months). No new relapses occurred during that period and IVIG treatment was temporally associated with clinical improvement and resolution of MRI lesions; the patient was still relapse-free 12 months after discontinuation of IVIG.

### Long-term outcome

At last follow-up, VA was impaired in at least one eye in 21/38 (55.3 %) patients with a history of clinical ON (median observation time 53.5 months, range 1-507) and around one third (14/38; 36.8 %) of all patients were either functionally blind at last follow-up in one eye or both or had a severe visual impairment (VA >0.1 and ≤0.5). Functional blindness (VA ≤0.1) in at least one eye was noted in 10/38 (26.3 %) patients, severe visual impairment but no functional blindness (VA >0.1 and ≤0.5) in 4/38 (10.5 %), moderate impairment (VA >0.5 and ≤0.75) in 2/38 (5.3 %), and mild impairment (VA >0.75 and <1.0) in 5/38 (13.2 %). Both optic nerves had been affected at least once at last follow-up (median observation time 54 months, range 1-394), either clinically or subclinically (i.e., based on MRI, VEP, fundoscopy, and/or OCT findings only) and either simultaneously or successively, in 35/42 (83.3 %) patients, while only one optic nerve had been affected in the remainder (median observation time 29.5 months, range 5-507).

Severe paresis was present at last follow-up in 1/28 (3.6 %) patients with a history of clinical myelitis (median observation time 41.5 months, range 6-102), moderate paresis in 4/28 (14.3 %), and mild paresis in 6/28 (21.4 %). Ambulation was impaired at last follow-up due to paresis and/or gait ataxia in 25 %.

If the total cohort is considered, VA was reduced at last follow-up in 23/47 (48.9 %) patients with available data (median observation time 49 months, range 1-507) and paresis was present in 14/48 (29.2 %) (median observation time 50.5 months, range 1-507).

### EDSS at last follow-up

**Fig. 12:**
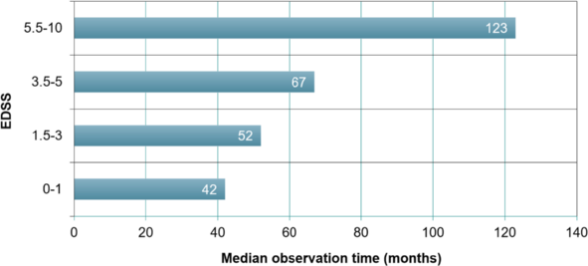
Increase in median EDSS scores with observation time in 47 MOG-IgG-positive patients

The expanded disability status scale (EDSS) was developed for use in classical MS and strongly focuses on ambulation deficits [47]. When interpreting EDSS results, it should be taken into consideration that complete bilateral visual loss corresponds to an EDSS score of just 4 and that patients with isolated ON can reach no higher scores. In accordance with that well-recognized underrepresentation of visual deficits – the main long-term sequelae in our patients (with functional blindness or severe visual loss present in 36 %) – EDSS scores were nominally low at last follow-up in most cases (median 2.5 [range 0-10] in the total cohort, *N* = 47, and 3 [range 0-10] among patients with relapsing disease, *N* = 40). A median EDSS ≥3.5 was reached after more than 60 months (Fig. 12). The median EDSS was 3 (range 1-10) among patients with an observation period of ≥100 months (*n* = 12) and 3.25 [range 1.5-10] among patients with an observation period of ≥120 months (*n* = 8). A higher median EDSS at last follow-up was noted in women (3, range 0 -10; *n* = 35) than in men (1, range 0-6; *n* = 12; *p* < 0.05) despite a longer median observation period in the male subgroup (72 months [range 1-127] vs. 50.5 months [range 1-507]).

### Survival rate

After a median follow-up period of 52 months (range 1-507), 49/50 (98 %) patients were still alive. One patient died from severe brainstem encephalitis leading to respiratory insufficiency 123 months after disease onset and after a total of 27 attacks, including attacks of ON, myelitis, encephalitis and/or brainstem encephalitis (see case 1 in part 3 of this article series [31]).

## Discussion

MOG-IgG-positive ON and myelitis are increasingly recognized as important differential diagnoses of AQP4-IgG-positive NMOSD. Here, we comprehensively analyzed the clinical, laboratory, radiological, and electrophysiological features of one of the largest cohorts of MOG-IgG-positive patients reported to date, as well as treatment responses and long-term outcomes. In our cohort, which was characterized by the longest observation time so far (mean 75 ± 46.5 months since onset, median 52 [1-507] months), the disease took a relapsing course in most cases. Attacks were often severe and characterized by substantial visual loss or by paresis with longitudinally extensive spinal cord inflammation. Many patients had radiological and/or clinical signs of brain and brainstem involvement. While a relatively favorable long-term outcome was noted in the majority of cases, the disease caused persistent severe visual impairment including unilateral blindness in more than one third of all patients with a history of ON, persistent mild to severe paresis or gait ataxia in almost 50 % of all myelitis patients, and was fatal in one patient due to recurrent brainstem attacks. Flare-ups after steroid treatment were noted in more than 40 % of cases, and even PEX was not always effective. In around 70 % of our patients, relapses occurred despite immunosuppressive therapy at least once. Given that genetic factors have been suggested to play a role in NMO, it is a potential strength of the present study that the cohort investigated here was genetically relatively homogeneous, with all patients except one being of Caucasian origin.

In this study, MOG-IgG were detected by means of new generation cell-based assays (CBA) employing recombinant full-length human MOG instead of enzyme-linked immunoassays, which are prone to both false-negative and false-positive results and which are no longer recommended for clinical routine diagnosis of MOG antibodies [10]; a CBA was also used for detection of AQP4-IgG [7, 8].

### Substantial phenotypic overlap with AQP4-IgG-positive NMOSD and MS

MOG-IgG-positive myelitis and ON showed a significant overlap with AQP4-IgG-positive NMOSD in clinical and radiological presentation, with more than 60 % patients with a history of both ON and myelitis meeting Wingerchuk’s 2006 criteria for NMO [28] and around a third of all patients fulfilling the revised 2015 criteria [29]. Even manifestations considered relatively typical for AQP4-IgG-positive NMOSD, such as medulla oblongata lesions and intractable nausea and vomiting or ON with involvement of the optic chiasm, were noted in some cases. Moreover, MS was initially suspected in more than a third of all patients, and every fourth patient with MOG-IgG-positive ON and/or myelitis presenting with brain and/or brainstem lesions met Barkhof’s criteria for MS, demonstrating a substantial phenotypic overlap between these two conditions.

With the discovery of AQP4-IgG [1, 6, 48], MOG-IgG [10], N-methyl-D-aspartate receptor-IgG [49], and a plethora of often non-paraneoplastic autoantibodies identified in acute CNS inflammation over the past decade [50–54], including in patients with primary or secondary demyelination, it becomes increasingly clear that not all patients presenting with relapsing CNS disease of putative autoimmune etiology have classical MS – even if they formally meet the ‘positive’ clinicoradiological criteria for MS [46]. MOG-IgG-positive patients in whom the disease starts with isolated brain or brainstem involvement are particularly challenging. Thus more and more importance attaches to carefully considering the ‘negative’ criterion of ruling out other diagnoses (“no better explanation”) included in the current diagnostic consensus criteria for MS [46].

Of note, 11 patients who met the clinicoradiological criteria for MS and 11/14 patients in whom a diagnosis of MS was initially suspected by their then treating physicians were negative for CSF-restricted OCB. Similarly, many patients with AQP4-IgG-positive NMO who were falsely diagnosed with MS in the past were negative for OCBs in a previous study [34]. This suggests that CSF analysis should be re-included in the diagnostic criteria for MS as an important tool to exclude alternative diagnoses, as previously recommended by us and others [55]. Moreover, 11/16 patients in whom MS had been initially suspected, later developed LETM lesions, which are not typically present in classical MS. In total, 15 out of the 16 patients were either negative for OCBs or had LETM lesions.

### Most patients have relapsing disease

The relatively long observation time is a particular strength of the present study, since it allows assessment of disease course and outcome in the long run. While previous studies with shorter observation periods (12 months in [13], 18 months in [11], 2 years in [12]) and smaller sample sizes (4 patients in [13], 9 in [11], 16 in [12]) suggested that MOG-IgG-positive patients might often have monophasic disease, our series demonstrates that most MOG-IgG-positive patients with ON or myelitis have a relapsing disease course. Moreover, a very short median time to first relapse of just 5 months was noted in this cohort, indicating an overall high risk of early relapse in MOG-IgG patients.

Given that (i) the observation period among ‘monophasic’ patients was significantly shorter than in the relapsing subgroup and below the median time to relapse in around one third of the ‘monophasic’ patients, (ii) the proportion of relapsing patients increased with observation time (Fig. 2), and (iii) the interval between the first and the second attack was long in some of the relapsing cases (>12 months in eight; up to 492 months), it is conceivable that some of the few ‘monophasic’ patients will develop further attacks in the future. A monophasic course of disease might thus be even less common than suggested here.

On the other hand, time since onset was >5 years at last follow-up in 3 patients in the monophasic group, all of whom were not treated with immunosuppressants, so the disease may in fact follow a monophasic course at least in small proportion of cases.

Similarly, the significantly shorter observation time since onset in patients with a history of ON but no myelitis or of LETM but no ON than in patients with a history of both ON and myelitis (i.e., NMO) suggests that the differences in presentation between these groups are probably an effect of observation time and that some of our patients with isolated ON may develop myelitis in the future and some of those with isolated LETM may develop attacks of ON. Indeed, disease had started with either isolated ON or isolated myelitis (rather than simultaneous ON and myelitis) in around three fourths of patients with a diagnosis of NMO at last follow-up. Importantly, myelitis occurred only after several ON attacks in some of these patients and ON only after several myelitis attacks in others. Similarly, disease starts with isolated ON or myelitis rather than simultaneous ON and myelitis in the vast majority of AQP4-IgG-positive patients [34].

These findings are highly important when it comes to deciding whether to treat MOG-IgG-positive patients or not. The frequently relapsing course observed in the present cohort indicates that prophylactic long-term immunotherapy should be considered in MOG-IgG-positive patients. Given that a relapsing course was also noted in 5/8 (63 %) patients with onset under the age of 18, this might possibly hold true also for children and adolescents. Studies systematically investigating the efficiency of long-term immunosuppression and/or immunomodulation in MOG-IgG-positive ON and myelitis are therefore strongly warranted. Moreover, given the lack of systematic long-term treatment data in MOG-IgG-positive disease, currently planned or ongoing treatment trials in NMO that include AQP4-IgG-negative patients should consider testing for MOG-IgG to allow subgroup analyses.

### Severe attacks and unfavorable long-term outcome are relatively frequent

Severe attack-related disability was noted in many cases, including tetraparesis in around 30 %, severe motor dysfunction with MRC grades ≤2 in around 25 %, pain -and/or dysesthesia in around 70 %, bladder and bowel disturbances in around 70 %, functional blindness in almost 75 %, bilateral optic nerve damage in 51 %, scotomas in 66 %, and brainstem encephalitis with, among other symptoms, ataxia, intractable nausea and vomiting or, of particular note, attack-related respiratory insufficiency in two patients, which was fatal in one. Importantly, long-term outcome was characterized by marked persisting visual impairment or blindness and/or significantly impaired ambulation in 40 %. Moreover, inflammatory damage was noted in the entire CNS, with the spinal cord, optic nerves, brainstem, diencephalon, cerebellum, and telencephalon affected in individual patients. These findings, together with the mostly relapsing course observed in our patients, underline that MOG-IgG-related CNS autoimmunity is a severe condition that requires consistent treatment and care.

Although a favorable outcome was noted in several patients, our findings do not support the notion that MOG-IgG seropositivity generally denotes a mild disease course [11, 13]. Again, previous studies reporting such findings may have been unintentionally biased by short observation periods and small sample size.

It should be taken into account in both epidemiological and therapeutic studies in the future that optic nerve damage is the leading manifestation of MOG-IgG-positive autoimmunity and that MOG-IgG-positive patients may present for many years or decades with isolated ON. The EDSS, which was developed for use in classical MS and which largely focuses on ambulation, may not sufficiently reflect the high degree of disability resulting from persisting visual loss in a substantial number of MOG-IgG-positive patients. Other scales of disability may need to be used in addition.

### IVMP was not always effective and flare-ups were frequent

In this context it is relevant that high-dose IVMP, though effective in many cases, was followed by only partial recovery or no recovery in 50 % of all treated attacks. Moreover, IVMP lead to only temporary improvement in 44 % of all patients at least once, resulting in flare-up of symptoms requiring repeat or ultra-high-dose IVMP therapy. In at least one case symptoms flared up not immediately after IVMP treatment but in a delayed fashion after tapering of subsequent oral steroid treatment. In some cases, even ultra-high-dose IVMP was ineffective or only transiently effective with a second flare-up occurring shortly after. Interestingly, in some patients IVMP was effective during initial attacks but not later in the disease course.

The occurrence of cerebral venous sinus thrombosis in one of our patients highlights the risks that repeat IVMP therapy and escalation to ultra-high-dose IVMP carry.

It is unknown why IVMP was effective during some attacks but not all. However, timing issues and differences in antibody titers, other immunological parameters (e.g., T cell activation), IVMP dosage, and previous or concomitant treatments might play a role.

Given the high frequency of flare-ups observed in our cohort, close clinical monitoring after acute attack therapy for MOG-IgG-positive ON and/or myelitis is recommended. Moreover, oral tapering of corticosteroid therapy as well as additional PEX treatment (see below) should be considered.

### MOG autoimmunity may underlie CRION in a subset of patients

Given the high proportion of patients with flare-up of ON after steroid withdrawal, i.e., of steroid-dependent ON, we propose that a subset of patients previously diagnosed as having CRION [56] may in fact have MOG-IgG-positive ON. Indeed, at least 3 of our patients had received a diagnosis of CRION before MOG-IgG was detected. Testing of larger cohorts of patients with CRION for MOG-IgG is highly warranted.

### PEX treatment was often followed by full or partial recovery

PEX was used in most cases as rescue therapy if steroids did not result in complete recovery; only in four patients was PEX used as first-line treatment for acute attacks. Of note, PEX treatment (as stand-alone therapy or following IVMP) was followed by complete or almost complete recovery in a substantial number of attacks (around 40 %). For example, in case 2 ON symptoms flared up twice after high-dose and subsequent ultra-high-dose IVMP therapy; only PEX ended the attack and was followed by complete recovery. The efficacy of PEX in this and other cases of MOG-IgG-positive ON and/or myelitis has potentially important pathophysiological implications, since it suggests a direct pathogenic role of the antibody. Interestingly, PEX treatment stopped the progression of dysesthesia in case 9, one of only 3 cases in which a slowly progressive (yet also relapsing) course of disease was noted.

However, as a limitation, it should not be overlooked that in almost 60 % of attacks treated with PEX, and thus in the majority of cases, only partial recovery was achieved, and in 2 cases there was no response to PEX. The variability in response to PEX may be linked to differences in PEX timing; MOG-IgG titers; intensity, extension, and site (e.g., ON vs. myelitis as seen in AQP4-IgG-positive NMOSD [35]) of inflammation; and, importantly, the number of PEX courses applied, which varied between 3 and 11 in the present cohort. Preliminary findings from our laboratory (S.J., unpublished data) show that AQP4-IgG and MOG-IgG may remain detectable even after five to seven plasma exchanges, raising the question of whether PEX treatment is discontinued too early in some cases. This is also supported by the early reoccurrence of attacks in cases 1 and 9 in part 3 of this series [31], just 1, 2 and 3 months after PEX. Alternatively, T cell-mediated mechanisms might play a more important role than antibody-mediated mechanisms in patients who do not sufficiently respond to PEX.

On the understanding that the use of PEX after IVMP implies previous IVMP failure, only incomplete recovery or no recovery at all was achieved in around 60 % of all attacks treated with IVMP (*N* = 147). Twenty-five of those attacks were subsequently treated with PEX, and full recovery was achieved in 40 % of them. This would suggest a beneficial role of PEX in MOG-IgG-positive patients with IVMP failure, similar to what has been observed in AQP4-IgG-positive NMOSD [35]).

The overall good response to escalatory PEX therapy, together with the risks associated with extensive cortisone pulse therapy as highlighted by the occurrence of sinus thrombosis with brain edema and seizures in case 2 might suggest that PEX treatment should be considered more often in patients with MOG-IgG-positive ON and/or myelitis. PEX may be considered as a substitute for escalatory ultra-high-dose IVMP therapy for severe attacks, particularly in patients who have responded well to PEX in the past. However, the observation of urosepsis in case 1 of part 3 [31] after several cycles of PEX illustrates that attention must be paid also to risks associated with PEX and IA, especially if those treatments are applied repeatedly and in combination with IVMP or IS treatment.

### Breakthrough attacks despite long-term immunotherapy

Similarly, long-term IS and IM treatments were not always effective in preventing further relapses. Almost 70 % of all patients treated with IS or IM drugs developed at least one attack during therapy. This included patients receiving AZA, MTX, NAT, IFN-beta, GLAT, rituximab, ofatumumab, and mitoxantrone. In case 6 at least 12 attacks of ON and myelitis occurred under various immunotherapies, and as many as 15 attacks occurred in case 1 in part 3 of this article series [31].

Complications of IS/IM therapy were rare in this cohort and included condylomata acuminata requiring surgical treatment, elevated liver enzymes under AZA treatment, and an allergic reaction to rituximab.

### AZA failure was associated with latency period and lack of cotreatment

AZA, which has been previously reported to be partially effective in NMO [57–59], including in AQP4-IgG-positive NMOSD [60], was the most commonly applied IS therapy in our cohort. However, more than 80 % of all AZA-treated patients experienced at least one attack while under therapy. As AZA has a latency period of 3-6 months during which cotreatment with oral steroids has been recommended [33], we analyzed the temporal pattern of AZA failure. Of 34 attacks during AZA treatment, 14 (41.2 %) took place during the first 6 months (11 during months 1-3 and 3 during months 4-6). Furthermore, 12 of those 14 attacks (85.7 %) occurred in patients (*n* = 6) not cotreated with oral steroids, PEX, or other immunosuppressants during that period. This suggests that AZA failure in MOG-IgG-positive patients may be caused in a substantial proportion of cases by the drug’s well-known latency in efficacy. Moreover, it may indicate that cotreatment with oral steroids during the initial 6 months of AZA treatment should not be abandoned in MOG-IgG-positive patients, provided contraindications have been excluded. However, it must be mentioned as a potential limitation that AZA was discontinued early in some patients after breakthrough attacks occurred, which may have introduced a considerable bias towards a higher proportion of attacks in the first 6 months. Larger studies are therefore needed before any treatment recommendations can be made.

Future studies on the efficacy of AZA and oral steroids as well as on that of oral steroids as stand-alone therapy in MOG autoimmunity should take into account that a recent retrospective analysis suggested a better response rate to high-dose azathioprine (2.5-3 mg/kg) than to standard treatment (1-1.5 mg/kg) in AQP4-IgG-positive NMOSD [58]. Whether such a high-dose regimen is also required in MOG-IgG-positive patients is currently unknown.

### Low relapse rate under MTX in most but not all cases

A recent study suggested that MTX might be effective in patients with AQP4-IgG-positive NMOSD [33, 61]. In our cohort, eight MOG-IgG-positive patients with ON and/or myelitis were treated with MTX, and exact data on attack dates were available from six. A lower relapse rate than in the total cohort and long attack-free intervals were observed in most MTX-treated patients. Based on these preliminary yet promising results, further retrospective studies seem warranted to assess the efficacy of MTX in MOG-IgG-positive ON and/or myelitis.

### Attacks related to initial rituximab infusion and reappearance of B cells

Rituximab treatment was followed by a clear reduction in relapse rate in three out of nine patients. In the six remaining patients, relapses occurred 2, 3, 4, 4, 7, 8, 8, 12 and 20 weeks after the first or second infusion (in one case despite PEX treatment 1 month earlier). This is reminiscent of the transient deterioration reported in some patients with AQP4-IgG-positive NMO after commencement of rituximab, which is associated with an temporary increase in BAFF and autoantibody levels [62, 63]. Another MOG-IgG-positive patient who experienced postinduction relapses (three within 3 months) has recently been described [63]. Whether cotreatment with steroids can prevent such events still needs to be explored.

Of note, one patient relapsed immediately after reappearance of B cells. This is similar to what has been observed in AQP4-IgG-positive NMO patients [64, 65] and suggests that (i) B cells should be closely monitored in MOG-IgG-positive patients treated with rituximab and (ii) treatment intervals should be short and doses high enough to prevent B cell reappearance. Rituximab has also been found to be effective in AQP4-IgG-positive NMOSD in some studies [65, 66], though not in all [67].

Ofatumumab is a fully human anti-CD20 monoclonal antibody which targets an epitope distinct from that of rituximab [68]. The marked reduction in relapse rate in the single patient treated with ofatumumab in this study is promising. However, more data are needed before any recommendations can be made. To the best of our knowledge this is the first report on ofatumumab both in MOG-IgG- and in AQP4-IgG-associated EM.

### Ongoing or increasing disease activity under IFN-beta

In the present cohort, 4 patients were treated with IFN-beta. All 4 showed ongoing or increasing disease activity. Although preliminary, these data suggest that IFN-beta, which has already been shown to be ineffective and to cause disease exacerbation in AQP4-IgG-positive NMOSD [69–72], may also be ineffective or even detrimental in MOG-IgG-positive patients. Given the substantial clinical overlap between MOG-EM and conventional MS, a condition often treated with IFN-beta, this would be of high clinical relevance. Larger retrospective studies evaluating the efficacy of IFN-beta in MOG-IgG-positive EM are therefore highly warranted.

### Preliminary data do not support use of GLAT or NAT

Like IFN-beta, GLAT is frequently used to treat patients with conventional MS. With the efficacy of GLAT being equivocal in two patients and eight breakthrough attacks having occurred in another three, the use of GLAT cannot currently be recommended in MOG-IgG-positive EM. Of note, GLAT has also been suggested to be of no clear benefit in patients with AQP4-IgG-associated NMOSD [36].

NAT, another drug shown to be beneficial in MS, could not prevent relapses in three MOG-IgG-positive patients in our cohort. While these preliminary data are not supportive of the use of NAT in MOG-IgG-positive ON or myelitis, systematic studies are certainly needed before definite conclusions can be drawn. NAT has also been found to be ineffective or even detrimental in patients with AQP4-IgG-positive NMOSD in recent studies [73–75]. Numerous relapses also occurred in a patient treated with mitoxantrone, another agent considered effective in conventional MS.

The failure of the MS therapeutics IFN-beta, GLAT and NAT in many of our patients supports the view that classical MS and AQP4- or MOG-IgG-associated disorders differ in terms of immunopathogenesis. This stance is further supported by the lack of OCB, a hallmark of conventional MS, in most of our MOG-IgG-positive patients as well as in most patients with AQP4-IgG-positive NMOSD [34, 76].

### MOG-IgG needs to be considered in children as well as in elderly patients

The median age at onset was around 30 years, which is similar to MS but differs from that in AQP4-IgG-positive NMOSD (~39) [34] by almost 10 years. However, the youngest patient in this cohort was just 6 years of age at onset and the oldest experienced his first attack at age 70, suggesting that MOG-IgG-positive ON and/or myelitis – just like AQP4-IgG-positive NMOSD [34] – can occur irrespective of age and need to be considered also in children and in the elderly.

All four patients with onset during childhood (at the ages of 6, 10, 12, and 12 years) initially presented with ON (bilateral in three), accompanied by myelitis in only one of them. Similarly, disease started with ON in the four oldest patients (onset at 58, 64, 66 and 77 years). Of note, 8/11 (73 %) patients with onset at age <20 had a recurrent disease course at last follow-up, which led to relevant disability in 5 of them (EDSS 3.5, 3.5, 6.0, permanent unilateral blindness, and VA of 0.2, respectively, at last follow-up), and in one of the two remaining patients observation time was too short to rule out relapsing disease. This would argue against the notion that MOG-IgG-positive ON in young patients is generally a monophasic disease and suggests that long-term immunotherapy (e.g., with IVIG if immunosuppressants are to be avoided) should be considered also in children and adolescents; however, larger studies are certainly needed. A recurrent course was also present in three of the four oldest patients in this cohort.

Interestingly, the time between onset and first relapse was extraordinarily long in one of our patients (see case 6 in part 3 of this series [31]), who had suffered from a first attack of ON at age 12, followed by an LETM attack 41 years later. Of note, two further patients reported events during childhood that are compatible with a first attack of MOG-IgG-positive EM (two episodes of bulbar movement pain, diplopia, and headache at ages 10 and 11 in case 23, and “neurogenic diabetes insipidus” at age 7 in case 22). As it remains unclear whether those early events were caused by the same disorder as the patients’ more recent complaints, which started 18.5 and 22 years later, they were not considered for statistical analysis.

With an age at onset of 70 years, patient 20 is, to the best of our knowledge, the oldest Caucasian MOG-IgG patient reported to date. The youngest patient described in the previous literature was just 1 year of age [77] and the oldest, a Japanese patient, 70 years [12] at onset.

Similarly, AQP4-IgG-positive NMOSD has been described both in children and in elderly patients [78–81]. Accordingly, MOG-IgG-positive EM is an important differential diagnosis of AQP4-IgG-positive NMOSD irrespective of age.

### Women are more often affected than men

Women outnumbered men by a factor of around 2.8 in this study. Female gender has also been identified as a risk factor for AQP4-IgG-positive NMOSD [82, 83]. However, a significantly higher preponderance of women (a male to female ratio of around 1:9) has been found in the latter condition in Caucasian patients [34]. The ratio found among MOG-IgG-positive patients in the present and previous cohort is more similar to that found in AQP4-IgG-negative NMOSD [34] and in classical MS [84]. Women might possibly be affected more severely than men as indicated by a higher median EDSS at last follow-up despite shorter median disease duration; however, confirmatory studies are needed to verify this finding.

### Attacks may occur during pregnancy and post partum

The effect of pregnancy on MOG-IgG-positive EM has not yet been systematically investigated. A recent study (*n* = 16) indicated that pregnancy may negatively influence the disease course of NMO; however, no data on the patients’ AQP4-IgG or MOG-IgG status were given [85]. The authors found a significantly higher attack rate in the first trimester after pregnancy and greater disability progression 1 year after delivery [85]. In another cohort [86], 14/40 AQP4-IgG-positive patients developed the first symptoms of NMOSD either during pregnancy (*n* = 3) or within a year after delivery or abortion (*n* = 11). While the ARR during pregnancy did not differ from that before pregnancy, it increased significantly during the first and second trimesters after delivery; moreover, 77 % of all deliveries were associated with post-partum relapses. In MS, pregnancy is thought to reduce the number of MS relapses, especially in the second and third trimesters; although attack rates tend to rise in the first 3-6 months post partum, no increased long-term disability has been found. In the present cohort, around a quarter of MOG-IgG-positive women experienced one or more attacks during pregnancy or post partum. Of special note, the disease started post partum in three of these patients. This could indicate that pregnancy- and/or delivery-related immunological changes may play a role both in triggering attacks and, possibly, in disease induction. However, given that most attacks in these patients as well as in the total female subgroup occurred irrespective of pregnancy and delivery, other risk factors may be more important. Based on these data, systematic prospective studies on the role of pregnancy and delivery in MOG-IgG-positive EM are warranted.

### Attacks may follow infection or vaccination

Attacks were preceded by infection in around 40 % of patients at least once, and disease started shortly after an infection in at least 11 cases and after vaccination in two cases. This is similar to AQP4-IgG-positive NMOSD, which has been reported to be preceded by infection in 20-30 % of cases [34, 87]. Acute infections are also thought to trigger clinical attacks in classic MS. However, the exact relationship between infection or vaccination and MOG-IgG-positive EM is unknown. While there is no evidence yet for molecular mimicry, it is conceivable that infection-associated immunological changes and/or blood-brain barrier disruption could promote CNS lesion formation. In AQP4-IgG-positive NMOSD, acute relapses are indeed associated with an elevated QAlb, which can be caused by structural barrier damage [76, 88]. QAlb was also elevated in around one third of MOG-IgG-positive patients in the present study. As a limitation, QAlb likely also reflect changes in the CSF flow rate [45]. It is of potential interest and deserves further investigation that the two post-vaccinal cases both occurred after vaccination against tetanus, diphtheria and pertussis. Of note, both patients developed relapsing disease. This is different from conventional postvaccinal ADEM, which is usually monophasic.

### CSF findings differ from MS but mimic AQP4-IgG-positive NMO

Examination of CSF harbors important potential for differentiating classical MS and MOG-IgG-positive EM but not MOG-IgG-positive EM and AQP4-IgG-positive EM: As in AQP4-IgG-positive patients [76], OCB and a positive IgG CSF/serum ratio, which are present in most patients with MS and which are thus considered a diagnostic hallmark of that disease, were missing in around 90 % of our MOG-IgG-positive patients. Moreover, OCB disappeared later in 2 out of the 6 only OCB-positive patients; by contrast, OCB are considered to remain stable for decades in MS [89]. Finally, neutrophil granulocytes, which are also present in AQP4-IgG-positive NMO [34, 76, 90] (as well as in bacterial meningoencephalitis [91]), were found in the CSF at least once in 64 % of cases, but are absent in classical MS.

Missing OCB or granulocytic pleocytosis should thus prompt physicians to challenge the diagnosis in patients with suspected MS and to consider MOG-IgG- or AQP4-IgG-positive encephalomyelitis.

### Subclinical evidence for dissemination in space

Electrophysiological evidence for optic nerve damage was present in at least 3 patients with no history of clinically apparent ON, and for spinal cord damage in at least 3 patients with no history of clinically apparent myelitis in our cohort. Similarly, supratentorial, brainstem, or cerebellar MRI lesions were present in 21 patients who had never shown clinical signs of encephalitis or cerebellitis but only of ON and/or myelitis. Finally, spinal cord MRI lesions were detected in 2 patients with ON but no history of clinical myelitis. This indicates that subclinical inflammation occurs in some cases and that clinical examination needs to be complemented by electrophysiology and MRI to assess the real extent of CNS inflammation in MOG-IgG-positive patients.

If not only clinical attacks are taken into account but also clinically silent lesions as detected electrophysiologically or by MRI, evidence for dissemination in space (defined as involvement of more than one of the following structures: optic nerves, spinal cord, supratentorial brain, brainstem, cerebellum) was present at last follow-up in 37/50 or 74 % of patients, compared with 26/50 or 52 % based solely on clinical grounds.

VEP and SSEP were not considered in the 1999 and 2006 diagnostic criteria for NMO, which required clinically apparent attacks of myelitis and ON, and are still not considered in the 2015 criteria for NMOSD [29]. Systematic studies on the potential prognostic, diagnostic, and therapeutic implications of pathological EP and MRI findings suggesting dissemination in space in patients with MOG-IgG-positive isolated ON or isolated myelitis are warranted. Evidence for subclinical optic nerve damage has also been reported in AQP4-IgG-positive NMOSD [92].

### Bilateral ON and simultaneous ON and myelitis are common at onset

More than 40 % of all patients with a history of both ON and myelitis at last follow-up presented with simultaneous myelitis and ON at least once, which is not different from what has been described in AQP4-IgG-positive NMO (42 % according to [34]). However, the frequency of simultaneous myelitis and ON at disease onset, i.e., as the initial presentation, was much higher in MOG-IgG-positive patients (23 % of all patients with a history of ON and myelitis) than in AQP4-IgG-positive NMOSD patients (6.7 % according to [34]; *p* < 0.03). Similarly, bilateral ON at onset was more frequent in MOG-IgG-positive patients with a history of ON (35 %) than in AQP4-IgG-positive NMOSD patients with a history of ON (14.3 % [34]; *p* < 0.04 ). Simultaneous ON and myelitis as well as bilateral ON at onset may thus be of diagnostic value and should prompt physicians to consider MOG-IgG testing.

### Short spinal cord lesions do not preclude MOG-IgG positivity

Spinal cord MRI lesions extending over three or more vertebral segments (so-called LETM) are considered a hallmark of AQP4-IgG-positive NMOSD, but are usually not found in classical MS. The presence of an LETM lesion in addition to clinical myelitis was also listed as a supportive criterion in the 1999 diagnostic criteria for NMO and one of three minor characteristics, two of which had to be present in addition to a history of ON and myelitis before a diagnosis of NMO could be made, in the 2006 criteria [28]. The association of MOG-IgG with LETM found in this and in previous studies is therefore of differential diagnostic importance.

However, two recent studies could demonstrate that up to 15 % of all MRIs of AQP4-IgG-positive patients show non-longitudinally extensive lesions [34, 93]. Similarly, lesions never exceeded two vertebral segments in 8 of our MOG-IgG-positive patients; in another 10 patients, at least one MRI showed only a non-LETM lesion but longitudinally extensive lesions were present in previous or later MRI examinations. The presence or absence of ‘short’ lesions in patients with AQP4-IgG- or MOG-IgG-positive myelitis is thought to depend, among other factors, on timing issues [94]. If MRI is carried out very early in the attack course or long time after an acute attack, lesions may be still evolving or be already in the process of resolution, respectively.

Similar to AQP4-IgG-positive myelitis, more than one lesion in the same MRI and swelling of the spinal cord were detected in many patients at least once. By contrast, necrotic lesions leading to spinal cord cavitation, as sometimes noted in AQP4-IgG-positive myelitis, were not reported in any of our MOG-IgG-positive patients.

### Lesions may affect the entire visual pathway

While retrobulbar optic neuritis was highly common among our MOG-IgG-positive patients, lesions affecting other parts of the optic pathway should be taken into consideration as well in MOG-IgG-positive patients presenting with visual symptoms. Many patients had signs of papillitis as detected fundoscopically; evidence for inflammation of the anterior part of the optic nerve was also found by MRI (Fig. 9). However, some patients presented with lesions in the chiasm (Fig. 9) and/or with longitudinally extensive ON (LEON) affecting both the anterior and the posterior portion of the optic nerve. Both LEON lesions and chiasmatic lesions were previously thought to be indicative of (AQP4-IgG-positive) NMOSD [29]. Our findings are in line with a recent Australian study that reported greater optic nerve lesion lengths in MOG-IgG-associated ON and AQP4-IgG-associated ON than in MS-related ON [95]. In a single patient, visual disturbances were associated with lesions within the optic tract (Fig. 9). Finally, some patients had occipital white matter lesions.

### Perioptic contrast enhancement warrants further investigation

As shown in Fig. 9, contrast enhancement was not only seen within the optic nerve but also in the perioptic nerve sheath and the immediately surrounding orbital tissue. This imaging pattern is of potential differential diagnostic relevance and, thus, deserves to be further investigated. In accordance with this finding, Kim et al. in a very recent study found perineural enhancement in 6 of 18 MOG-IgG-positive patients [96]. As is the case with other MRI features, it is likely that the presence or absence of that phenomenon depends on disease and treatment status: more than one third of all MRIs without perioptic enhancement in our cohort were performed during remission, and some of the remaining patients had been treated with high-dose IVMP before the MRI was performed.

### Coexisting autoimmunity is rare in MOG-IgG-positive patients

Coexisting autoimmune disorders are present in more than one third of AQP4-IgG-positive NMOSD patients [34]. By contrast, only around 9 % of our MOG-IgG-positive patients had a coexisting autoimmune disorder (2 × RA, 1 × Hashimoto thyroiditis, 1 × Grave’s disease). Systemic lupus erythematosus, Sjögren syndrome, and myasthenia gravis, which are common in AQP4-IgG-positive NMOSD [97–102], were absent in all of our MOG-IgG-positive patients. This is in line with a previous study that reported a lower frequency of concomitant autoimmune disorders in ‘AQP4-IgG-seronegative’ NMOSD patients [34]. Interestingly, first symptoms of anti-TPO-, anti-thyreoglobulin-, anti-TSH receptor-associated hyperthyreosis appeared just seven weeks after the first attack of MOG-IgG-positive myelitis in one of our patients, suggesting that MOG autoimmunity might have been part of a broader immune dysregulation in this case.

### Nosological issues

The 2015 diagnostic consensus criteria for NMOSD demand that “alternative diagnoses” should be excluded [29]. However, it remains unclear whether MOG-IgG-positive ON or myelitis should be considered an “alternative diagnosis” or not [103–105]. AQP4-IgG-positive and MOG-IgG-positive EM differ in terms of target structures (astrocytes vs. oligodendrocytes) and, accordingly, immunohistopathology [18–20], but MOG-IgG is not explicitly mentioned as an exclusion criterion. This mainly reflects the fact that at the time the criteria were developed, data on MOG-IgG-positive patients were still scarce.

In the present study, only around one third of all MOG-IgG-positive patients met the clinical and radiological criteria for “NMOSD without AQP4-IgG” [29]. If MOG-IgG is not considered an exclusion criterion for NMOSD, this would result in a subset of MOG-IgG-positive patients being considered eligible for clinical studies and treatment trials, while others would be excluded based solely on phenotypic presentation and despite all of these patients belonging to the same immunopathogenetically defined disease spectrum. This could introduce a relevant inclusion bias given that almost all of the patients who met the criteria had a history of ON and LETM (15/16 or 94 %), while most of those who did not meet the criteria had isolated ON or isolated LETM at last follow-up (27/34 or 79 %). Conversly, inclusion of MOG-IgG-positive patients in NMOSD cohorts (which are predominantly AQP4-IgG-positive) would introduce bias as well.

We therefore believe that confirmed MOG-IgG seropositivity should be considered an exclusion criterion for NMOSD and that the term ‘NMOSD’ should be restricted to AQP4-IgG-positive patients and, possibly, double-seronegative patients meeting the criteria for ‘AQP4-IgG-negative NMOSD’ [29].

There are two potential limitations to such an approach. First, using MOG-IgG seropositivity as an exclusion criterion for NMOSD would require providing a reference assay for MOG-IgG testing with excellent specificity as established in an appropriately controlled multicenter setting. Alternatively, however, diagnostic criteria for MOG-IgG-positive NMOSD based both on serological and on supporting clinicoradiological criteria could be established in analogy to the current consensus criteria for NMOSD. Second, so-called ‘double-positive’ patients, i.e., patients positive for both AQP4-IgG and MOG-IgG, would pose a diagnostic dilemma. However, such patients should in any case be excluded from clinical trials as they may have two immunopathophysiologically distinct diseases. Moreover, ‘double-positive’ patients seem to be extremely rare (see part 1 of this article series [30] and Table 4 in [17]) and the few cases reported so far worldwide have not been independently confirmed.

### Limitations

We acknowledge some obvious limitations of our study. First, the study design was retrospective, as in all previous studies in the field, and a high number of neurological centers were involved. However, due to the low prevalence of the condition, prospective single-center studies including sufficiently large numbers of patients are impracticable. Moreover, the multicenter design of this study, which included 11 academic centers, reduces the risk of referral bias, which was acknowledged as a possible limitation by the authors of previous large single-center studies in the field of NMO [28, 106]. Moreover, reliable assays for detecting MOG-IgG have only recently been developed; accordingly, only retrospective long-term data are currently available. Second, patients with a benign or monophasic long-term course are less likely to be admitted to hospital and might thus be under-represented in the present cohort. However, this type of potential bias is inherent in hospital-based studies and cannot be completely avoided. It is important in this context that all centers involved in the present study also have specialized outpatient clinics for patients with neuroinflammatory conditions and that participants were recruited among both inpatients and outpatients. Moreover, the threshold for admission is low in Germany and Italy, where public healthcare is free. Third, MOG-IgG has also been reported in patients with conditions classified as ‘ADEM’ based on clinical and radiological features, especially in children [107]. Such cases were not systematically included in the present cohort, which focused on patients with ON and/or myelitis. Given that our study found a relapsing disease course in most patients with MOG-IgG-positive CNS inflammation and that attacks did not develop until years after initial presentation in some cases, systematic follow-up studies on patients previously diagnosed with MOG-IgG-positive ‘ADEM’ seem warranted to confirm that rare association.

## Conclusions and outlook

In summary, our study demonstrates that MOG-IgG-associated ON and myelitis frequently follow a relapsing course and result in severe and/or persisting disability in a substantial number of cases. Functional blindness due to optic nerve damage is the most common disabling sequela. In addition to tetra- or paraparesis, dysesthesia and pain are common symptoms in patients with myelitis. Some patients experience mild attacks with purely sensory symptoms that may not be accompanied by marked MRI or electrophysiological changes. Although in our cohort most patients with MOG-IgG-positive myelitis had LETM, non-longitudinally extensive lesions were found on a number of MRI examinations and thus do not preclude the diagnosis. Coexisting clinical or radiological evidence for brain, brainstem, or cerebellar involvement is frequent and may be extensive in some cases. Brainstem symptoms may include intractable nausea and vomiting as well as life-threatening or fatal respiratory complications. As in AQP4-IgG-positive NMOSD, CSF examination reveals mostly mild pleocytosis (partly with neutrophils) and, in contrast with MS, no evidence of intrathecal IgG synthesis in the vast majority of cases. Treatment of acute attacks with IVMP and PEX was effective in many patients, and immunosuppressive therapy was often followed by relapse-free intervals; however, failure of acute and long-term treatment and, subsequently, rapid accumulation of disability was noted in several cases. Of particular note, flare-up of symptoms after discontinuation of IVMP for treatment of an acute attack is frequent in MOG-IgG-positive patients. Full recovery was achieved by PEX in some cases, including patients showing IVMP failure. Breakthrough attacks in AZA-treated patients occurred particularly during the latency period of AZA and in patients not cotreated with oral steroids. MTX was identified as a potentially effective treatment in MOG-IgG-positive ON and/or myelitis. IFN-beta was used in rare patients misdiagnosed with classical MS and was associated with an increase in disease activity. Rituximab was effective in some patients, but new attacks occurred within a few weeks after the first infusion in a subset of cases, similar to what has been reported in AQP4-IgG-positive NMOSD. Our series, which includes some of the youngest as well as the oldest Caucasian MOG-IgG-positive cases, demonstrates that MOG-IgG positivity should be considered in patients presenting with ON or myelitis of unknown origin irrespective of age. Women are affected more often – and possibly more seriously – than men. Coexisting autoimmunity in MOG-IgG-positive NMOSD seems to be rare compared with AQP4-IgG-positive NMOSD. A substantial overlap in clinicoradiological presentation both with AQP4-IgG-positive NMOSD and with classical MS was found, and many patients were initially diagnosed with MS. While some patients with MOG-IgG-positive ON and/or myelitis meet the 2015 international diagnostic criteria for NMOSD, others do not; this is problematic from a nosological point of view, assuming that the same immunopathogenesis underlies all MOG-IgG positive cases. Several clinical and radiological features hitherto thought to be typical for AQP4-IgG-positive NMO, such as longitudinally extensive spinal cord lesions, lesion location in the central portion of the spinal cord, longitudinally extensive optic nerve lesions, lesions involving the optic chiasm, area postrema lesions, intractable nausea and vomiting, and thalamic lesions, or for MS, such as INO or periventricular, subcortical, juxtacortical, and callosal white matter lesions, were present in some of our MOG-IgG-positive patients. Similar to AQP4-IgG-positive NMOSD and to MS, disease onset or relapse was preceded by infection or vaccination in several cases. Around 30 % of all the women in our cohort who gave birth at least once developed attacks during pregnancy or post partum.

Our findings from a predominantly Caucasian cohort strongly argue against the notion that MOG-IgG denotes a milder and usually monophasic variant of NMOSD, as suggested by previous, smaller cross-sectional studies with shorter observation periods. Given the relapsing and often severe disease course of MOG-IgG-positive ON and myelitis, the use of long-term immunosuppressive treatments in this condition should be considered. Prospective multicenter studies and treatment trials in MOG-IgG-positive EM will be difficult to perform due to the rarity of the condition but are highly warranted.

## Appendix

Due to the novelty and rarity of the disorder, large and comprehensive case series illustrating the broad and heterogeneous spectrum of clinical manifestations, disease courses, and radiological presentations in MOG-IgG-positive encephalomyelitis are lacking. In this appendix, we provide detailed reports on 28 cases of MOG-IgG-positive ON and/or myelitis. We believe that case reports can draw a more vivid ‘real-life’ picture of this rare disorder than statistical analyses alone. For the reader’s convenience, the most important findings are briefly summarized and discussed in a comment at the end of each report. Additional reports are to be found in the “Case reports” section in part 3 of this article series [31].

### Contents

I. MOG-IgG-positive NMO without brain involvement (cases 1-5)

II. MOG-IgG-positive NMO with brain involvement (cases 6-10)

III. MOG-IgG-positive recurrent ON (cases 11-18)

IV. MOG-IgG-positive monophasic ON (cases 19-24)

V. MOG-IgG-positive recurrent LETM (case 25)

VI. MOG-IgG-positive monophasic LETM (cases 26-27)

VII. MOG-IgG-positive postvaccinal NMO (case 28)

### I. MOG-IgG-positive NMO without brain involvement

*Case 1 – Recurrent LETM and ON resulting in persisting unilateral functional blindness and optic nerve atrophy.* This Caucasian woman first presented with ON of the right eye in 08/2004 at age 40. Symptoms completely remitted after IVMP treatment. Since then, at least seven relapses of unilateral ON have occurred, which have successively affected both eyes and only partially responded to IVMP or IVMP and PEX. In addition, she experienced two attacks of myelitis, one with paraparesis and bladder and bowel dysfunction (04/2009) and the other with gait ataxia and sensorimotor paraparesis (08/2011); spinal MRI showed LETM lesions extending from C5 to Th2 (04/2009) and at T5 and T11 (08/2011), respectively, with Gd enhancement. Multiple brain MRI examinations were normal or showed only a few non-specific lesions not meeting the Paty or Barkhof criteria for MS; in addition, atrophy of both optic nerves was noted. Electrophysiology revealed prolonged P100 latencies in both eyes; later potentials were lost. While CSF was negative for OCB, very mild pleocytosis (6 cells/μl) was noted. The patient was given AZA (150 mg/d) between 01/2010 and 08/2010, and MTX was commenced in 08/2010. During treatment with AZA, three ON attacks took place; one attack of myelitis and two of ON occurred within 8 months despite treatment with MTX. At last follow-up, the patient had VA of 0.1 in the left eye (and 1.0 in the right eye) but no paresis.

Comment: This case – characterized by ON and LETM, mild pleocytosis, no OCB, and a normal MRI at onset – illustrates how similar the clinical presentation in MOG-IgG-positive patients can be to that of AQP4-IgG-positive NMO. In fact, a substantial proportion of patients in this cohort met Wingerchuk’s 2006 criteria. Of further note, 10 attacks occurred within only 85 months in this patient. Overall, the disease took a relapsing course in 80 % of our patients. Moreover, as observed in many cases, acute treatment with IVMP and PEX and long-term immunosuppressive treatment were both only partially effective, resulting in a poor clinical outcome with functional blindness in one eye at last follow-up.

*Case 2 – Recurring LETM and IVMP-refractory ON in a young girl, complete recovery after PEX.* This young Caucasian woman experienced a first attack of myelitis in 06/2012 at age 17 with hypesthesia ascending from both feet to level T4, followed by predominantly left-sided paraparesis and urinary incontinence. Spinal MRI in 08/2012 demonstrated an LETM lesion extending from T3 to T6 with Gd enhancement (no swelling or necrosis documented). CSF WCC, OCB, QIgG, and total protein were normal. After IVMP (5 × 1000 mg) symptoms remitted except for a mild foot flexor paresis. AQP4 antibodies were negative.

A first relapse of myelitis occurred 1 year later, leading to hemihypesthesia predominantly of the left arm and torso. No MRI was performed. OCB were again negative. 5 × 1000 mg IVMP resulted in only partial recovery (EDSS in remission 3.0). AQP4-IgG was again absent, but MOG-IgG, which had not been previously tested, were positive.

In 07/2014, she suffered a first episode of unilateral ON with painful eye movement, blurred vision, and a small scotoma. VEP demonstrated prolonged P100 latency (amplitudes not reported). IVMP (5 × 1000 mg) resulted in transient full recovery. However, after just 2 weeks symptoms flared up again, with similar VEP results but an enlarged scotoma. Again, symptoms remitted completely after treatment with IVMP (3 x 2000 mg), only to recur after another 2 weeks. In between the first and second re-flare, thrombosis in the patient’s superior sagittal sinus occurred, most likely linked to extensive cortisone pulse therapy. This was associated with two symptomatic epileptic seizures probably due to focal edema. The second re-flare was therefore treated with five cycles of PEX, which resulted in complete remission. While VEP showed a prolonged P100 latency (140 ms) in the affected eye during relapse, VEP were normal at last follow-up in 03/2015.

Comment: As in patient 1, the disease took a severe, recurring course, though in this case final outcome was good. Of particular note, IVMP was effective during the first attack but not or only partially during subsequent attacks. Flare-ups after initial response to IVMP were noted in several other patients, too. In this case, only therapy escalation to PEX resolved the patient’s attack-related symptoms. The occurrence of sinus thrombosis with brain edema and seizures illustrates the risks of extensive cortisone pulse therapy.

*Case 3 – Simultaneous bilateral ON and LETM in a patient with rheumatoid arthritis; recurrent myelitis; complete recovery.* A 48-year-old woman with an 18-month history of suspected seronegative RA, which had been treated with oral MTX 15 mg per week starting in March 2009, developed bilateral pain on eye movement and severe visual loss in March 2010. Her personal and family history was otherwise unremarkable. A few days later, she additionally developed bilateral weakness of her legs which progressed to severe tetraparesis. Furthermore, she developed numbness of her trunk and legs starting at level T3, severe disturbances of micturition (residual urine 400 mL) and defecation, and severe back pain which was pronounced on head movement. Spinal MRI in an external hospital showed an LETM lesion extending from C2 to T4. Cranial MRI showed no inflammatory lesions but an old right-sided anterior infarction. A detailed search for sources of embolism was negative. CSF analysis revealed 73 leukocytes/μl and moderate blood-CSF barrier dysfunction, while OCB were negative. Both vasculitis screening including antiphospholipid antibodies and AQP4 antibodies were repeatedly negative, as were extensive infectiological investigations. The symptoms gradually improved during initial intravenous treatment with ceftriaxone, ampicillin, and aciclovir. Subsequent high-dose intravenous steroid pulse therapy over 5 days accelerated the remission of symptoms. When the patient first presented to our department 3 weeks after her symptoms had started, vision had normalized and walking was already possible without assistance. VEP amplitudes were reduced bilaterally and the P100 latency was slightly prolonged on the left side (121 ms). Tibialis SSEP were normal and MEP to the tibial anterior muscles showed bilateral slight prolongation of the central conduction time. The patient continued MTX treatment. In August 2010, a detailed neurological examination was unremarkable apart from mild proximal paresis of her right leg, not to be explained by the old anterior infarction on the same side. VEP, SSEP, and MEP were normal. The patient was then seen at half-yearly, later yearly intervals without developing further symptoms until September 2013, when she presented with acute painful numbness of her legs and lower trunk starting at level T10 and a positive Lhermitte’s sign. Cervicothoracic and cranial MRI did not show new or enhancing lesions. CSF analysis revealed 17/mm^3^ leukocytes and no evidence of a disturbed blood-CSF barrier function; isoelectric focusing again did not show CSF-specific OCB. Somatosensory and visual evoked potentials were normal. High-dose corticosteroid pulse therapy induced full remission of the symptoms. Cranial MRI in 07/2015 still did not show inflammatory lesions; cervicothoracic MRI in 08/2015 was unchanged. The patient was last seen in 08/2015 without having developed further new symptoms. She still continues treatment with MTX.

Comment: This patient’s initial presentation with simultaneous LETM and bilateral ON was considered typical of NMO for many decades. However, recent studies have demonstrated that AQP4-IgG-positive NMO starts with unilateral ON in the majority of cases; by contrast, simultaneous ON and myelitis as well as bilateral ON at onset have been found to be more common among ‘seronegative’ patients with NMO [34]. In the present study, the disease started with simultaneous ON and myelitis (with or without brainstem involvement) in 10 %, and the initial attack affected both eyes in 41 % of all patients with ON at onset, which corresponded to 30 % of all patients. Both the severity of the patient’s presenting symptoms and the promptness as well as the nearly complete remission observed in this case are remarkable. Of particular interest, the patient’s first relapse occurred while on treatment wth MTX for RA. In total, two attacks occurred within 6 years of MTX treatment. Relatively low relapse rates under treatment with MTX were also observed in cases 3, 6, and 13 described here as well as in cases 6 and 12 in part 3 of this series [31].

*Case 4 – Simultaneous myelitis and bilateral ON with complete recovery in a child.* This 10-year-old Caucasian girl experienced back pain and, within 10 days, developed intention tremor of the upper extremities and complete urinary retention. Neurological examination showed brisk knee jerks without Babinski’s sign and urinary retention, but was otherwise unremarkable. While cranial MRI was normal, axial spinal cord imaging showed two small, non-contrast-enhancing T2-hyperintense lesions in the cervical cord with normal signal in the remaining spinal cord including the conus. Ophthalmological assessment revealed mild bilateral ON with impaired color perception and mild papilledema of the left eye and slightly delayed P100 latencies elicited from both eyes. CSF analysis disclosed 179 cells/μl with a high proportion of neutrophil (69 %) and eosinophil granulocytes, mild blood-CSF barrier dysfunction, marginal intrathecal IgM synthesis, and CSF-restricted OCB. Serology and PCR testing ruled out infection with herpes simplex type 1, type 2, and type 6, varicella zoster virus, enterovirus, arbovirus, *Borrelia burgdorferi*, and *Treponema pallidum*. CSF cultures for bacterial microorganisms were also negative. The serum tested negative for AQP4-IgG, ANA, and ANCA. CRP was normal. MOG-IgG was present at a titer of Treatment included short-term antibiotics (ceftriaxone) followed by a 5-day pulse of high-dose IVMP and prompted rapid resolution of symptoms. At last follow-up 26 months after onset the patient did not report any residual symptoms; P100 latencies elicited from both eyes were slightly delayed.

Comment: This case – together with cases 8 and 12 as well as case 6 in part 3 of this series [31] – highlights that MOG-IgG-related NMO may begin as early as in childhood or adolescence. As in case 3, the patient presented with bilateral ON at onset and with simultaneous myelitis and ON, two presentations that were previously shown to be more common in AQP4-IgG-negative patients with NMO [34]. Of note, the patient’s spinal cord lesions did not extend over three or more vertebral segments. ‘Non-longitudinally extensive’ lesions have recently been described in two independent studies [34, 93] also in patients with AQP4-IgG-positive NMO; accordingly, LETM lesions are no longer listed among the new, revised 2015 criteria for AQP4-IgG-positive NMOSD [29]. It is of interest that mixed but predominantly neutrophilic pleocytosis was present in this patient; CSF neutrophils were also noted in 21 other patients with CSF pleocytosis and available data. Neutrophil granulocytes were shown to be present also in around 50 % of CSF samples in AQP4-IgG-positive NMOSD during acute attacks [76].

*Case 5 – Recurrent ON and purely sensory myelitis; good recovery.* This Caucasian man developed unilateral ON at age 22 with complete recovery after high-dose IVMP therapy. Lumbar puncture (LP) revealed CSF pleocytosis (128 white cells/μl) and blood-CSF barrier dysfunction. Brain MRI was normal except for optic nerve swelling and Gd enhancement. VEP demonstrated a delayed P100 latency but normal amplitudes. A second ON attack occurred 10 months later but symptoms again remitted completely following IVMP therapy. Over the following 117 months, two more attacks of unilateral ON with complete or almost complete recovery after IVMP occurred, and one attack of myelitis with purely sensory symptoms (including pain/dysesthesia) and bladder bowel dysfunction. Spinal cord MRI showed a short lesion (one vertebral segment) without swelling or Gd enhancement. IVMP treatment resulted in partial recovery. Serum AQP4-IgG was negative, but MOG-IgG was detected at a serum titer of 1:1280. At last follow-up, an EDSS of 1.5 and normal VA was documented. No relapses have occurred so far under treatment with rituximab, which was commenced 2 months before the time of writing.

Comment: This case again illustrates that lesions in MOG-IgG-positive myelitis are not always longitudinally extensive and often exclusively cause sensory symptoms, including pain and dysesthesia. Of note, the latter symptoms were the most common manifestations of myelitis in this cohort, having being present in around 70 % of all patients at least once.

### II. MOG-IgG-positive NMO with brain involvement

*Case 6 – LETM and 13 attacks of ON resulting in unilateral blindness; white matter and callosal lesions.* This Caucasian woman had a first, left-sided attack of ON at age 47, with visual loss and retro-orbital pain. MRI showed a longitudinally extensive Gd-enhancing optic nerve lesion on the left side and a clinically inapparent short, Gd-enhancing lesion in the posterior part of the prechiasmatic segment of the optic nerve which extended into the optic chiasm. Symptoms completely remitted following high-dose IVMP treatment. Within 4 months she developed transverse myelitis with thoracic pain, sensory impairment of the abdomen and legs, and urinary incontinence along with blurred vision affecting the other, previously clinically unaffected eye, yet retained high-contrast VA. Spinal MRI revealed two T2 hyperintensities in the thoracic cord; one of these lesions extended over three spinal segments and was accompanied by marked cord swelling and intense contrast enhancement. Cranial MRI showed several non-Gd-enhancing T2-hyperintense lesions in the subcortical supratentorial white matter not involving the corpus callosum. On neurophysiological testing, tibial nerve SSEPs had normal latencies, VEP amplitudes were bilaterally reduced, and P100 latencies were delayed on the left side. The CSF disclosed 14 cells/μl (mostly lymphocytes with a small number of neutrophils) and a normal protein profile without blood-CSF barrier dysfunction and no CSF-restricted oligoclonal IgG bands. The serum tested negative for ANA, anti-ds-DNA-Ab, extractable nuclear antigens (ENA), ANCA, and anti-phospholipid Ab; complement components C3 and C4 were also not altered. High-dose IVMP treatment followed by oral tapering of corticosteroids prompted almost complete recovery of clinical symptoms with mild dysesthesia and restlessness of the lower extremities.

Over the following 12 years, the patient experienced 12 further attacks of mostly unilateral ON, which emerged at a frequency of once or twice per year and were not affected by various long-term immune therapies: 2 of the 12 relapses occurred under treatment with i.m. IFN-beta-1a (1 and 4 months after first injection), 1 under s.c. IFN-beta-1a (less than 2 months after first injection), 1 under s.c. IFN-beta-1b (after 2 months), none under GLAT (6 months), and 1 during a short course of AZA (4 months; no cotreatment with oral steroids). Three PEX cycles (3-5 exchanges each) could also not prevent bilateral deterioration of VA due to recurrent ON with almost complete unilateral blindness and severe contralateral visual loss (0.4 on last follow-up). Of note, two relapses of ON occurred a few weeks after the first and the second infusion of rituximab, respectively. A further relapse of ON took place 12 months after initiation of rituximab therapy and thus probably after reconstitution of B cells. Treatment with IFN-beta-1a, IFN-beta-1b, and GLAT had to be stopped prematurely because of leukopenia; AZA was stopped due to elevated liver enzymes.

Some stabilization of ON frequency was achieved following prolonged therapy with MTX and long-term maintenance treatment with oral corticosteroids (combined with ciclosporin for a period of 7 months); the patient experienced only two further attacks of ON within almost 7 years after implementation of these regimens.

Repeat MRI 2 years after the initial ON revealed numerically stable brain lesions and complete regression of cord hyperintensities; however, after another 2 years, the brain lesion load had increased, with some lesions now involving the corpus callosum and infratentorial white matter tracts. Follow-up laboratory analysis revealed a normal CSF cell count with mildly elevated neutrophils (6 %) and protein profile with negative OCB. Serology for AQP4-Ab was negative.

Comment: This case once more illustrates that MOG-IgG-positive NMO is not always a mild and monophasic disease but can take a relapsing course with poor long-term outcome. Here, the disease caused functional blindness in one eye and marked visual loss in the other eye despite IVMP therapy for acute attacks and various immunotherapies. Of particular note and similar to case 12 described below and case 5 in part 3 of this series [31], INF-beta treatment was paralled by several relapses. By contrast, long-term treatment with MTX in combination with oral steroids was followed by significant stabilization.

*Case 7 – Recurrent ON and pregnancy-related LETM with permanent functional blindness in one eye; white matter lesions.* This female Caucasian patient developed a first episode of unilateral ON in 2005 at age 31 with painful eye movement and blurred vision making reading impossible. Several relapses of unilateral ON occurred up to 2013, all of which responded well to IVMP (5 × 1000 mg) with good partial or full remission of VA. In 04/2013, at which time she was 6 weeks pregnant, she developed numbness of both hands, later accompanied by intense pain of her right arm and shoulder. MRI in 05/2013 revealed an LETM lesion in the cervical spinal cord extending over five vertebral segments. In 12/2013, i.e., only a few weeks after delivery, severe bilateral ON occurred, starting in the left eye and shortly afterwards affecting the right eye. Treatment with IVMP (3 × 1000 mg) and therapy escalation with 2000 mg IVMP 2 weeks later did not result in any significant improvement of the patient’s VA (left eye 0.025 in 02/2014). Following the start of rituximab in 02/2014, very slow subjective improvement of visual function was noted. AQP4-IgG and a broad panel of other anti-neural autoantibodies were negative. MOG-IgG were positive in two samples. Brain MRI was normal at onset but showed inflammatory white matter lesions later. LP revealed pleocytosis (37 cells/μl) but no OCB.

Comment: Of particular note, two attacks in this patient occurred during pregnancy or shortly after delivery, respectively. Similarly, attacks occurred 1.5, 3 and 8 months after delivery in case 12 in part 3 of this series [31] and in cases 23 and 19 described below, respectively. One further patient experienced at least two attacks during pregnancy (see case 13 in part 3 [31]). An increase in relapses post partum has been reported in MS [108], and a negative influence of pregnancy on the disease course has been suggested in NMO as well [85, 86]. On the other hand, disease onset preceded pregnancy by more than 8 years in our patient and several relapses had occurred before the first pregnancy-related attack. A simple coincidence thus cannot be ruled out. Note the poor outcome in this patient: While IVMP was initially effective, both high- and ultra-high-dose IVMP therapy failed to restore vision later on, resulting in permanent visual loss in the left eye.

*Case 8 – LETM and recurrent bilateral ON with transient blindness but full recovery in a young boy, ventricular lesion.* This young Caucasian male patient first presented in September 2006 at age 12 with an attack of bilateral ON with blurred vision. The symptoms remitted partially after IVMP pulse therapy.

Six weeks later, he developed headaches and tetraparesis. MRI showed a supratentorial lesion at the anterior horn of the right lateral ventricle and a cervical lesion extending from C2 to C6 level with Gd enhancement and swelling. ADEM was suspected. Extensive laboratory examinations did not reveal any infectious or rheumatological cause. Family history was negative for relevant autoimmune diseases. LP showed a normal CSF WCC, normal CSF protein, glucose, and lactate levels, and negative OCB. After IVMP therapy, vision and muscle strength returned to normal.

The third attack occurred in 03/2012 with almost complete loss of vision of the right eye. LP revealed a normal CSF WCC, normal CSF protein concentration, and negative OCB. AQP4 antibodies were negative. Cerebral MRI showed Gd enhancement of the optic chiasm. VEP revealed bilaterally delayed P100 latencies with normal amplitudes. OCT demonstrated bilateral temporal thinning of the retinal nerve fiber layer (RFNL). No clinical signs or symptoms of myelitis were present, but MRI showed a T2 lesion at C3, spanning around one and a half vertebral segments, as well as diffuse lesions extending from level C4 to C7 and from level T7 to T9 with Gd enhancement and swelling. After treatment with IVMP, symptoms remitted completely. MS with an opticospinal focus or NMO was suspected and treatment with GLAT was initiated.

Spinal cord MRI in 09/2013 showed remission of the cervical lesions with a remaining small lesion at C2. At last follow-up in 06/2015, after 36 months of treatment with GLAT, the patient did not report any further clinical relapses and had only mild clinical deficits (EDSS 1).

Comment: While bilateral ON and myelitis did not occur strictly simultaneously in this patient, the interval between the two events was just 6 weeks. Similarly, a very short median time to first relapse (5 months) was found in the total cohort. This suggests that patients presenting with a first attack of MOG-IgG-positive ON or myelitis should be closely monitored and treated early. The severe symptoms noted during acute ON (almost complete unilateral blindness) contrast remarkably with the complete remission achieved after IVMP in this case. Note the young age at onset in this and in three other patients (cases 4 and 12 here and case 6 in part 3 of this article series [31].

*Case 9 – Bilateral ON and subsequent LETM with late onset and only partial recovery, callosal lesions on MRI.* This Caucasian woman developed bilateral optic neuritis in 11/2009 at age 64 with spontaneous complete remission within 6 weeks but subsequently prolonged P100 latencies. From 01/2011, she complained of increasing dysesthesia in both forearms and impaired ambulation. Spinal MRI in 05/2011 showed two cervical contrast-enhancing lesions. LP revealed identical OCB in CSF and serum (mirror pattern) and a normal cell count. AQP4 antibodies were negative. Except for ANA (1:160), no other concomitant autoantibodies were detected. Treatment with IVMP in 05/2011, 07/2011, and 09/2011 did not result in significant improvement of the still increasing dysesthesia. In 10/2011, spinal MRI showed diffuse hyperintensity ranging from C2 to C6 with Gd enhancement. Cranial MRI revealed numerous non-specific T2 hyperintensities considered to reflect microvascular lesions due to a long history of hypertension. In 12/2011 MOG-IgG was tested for the first time and was positive at a titer of 1:1280. After 11 courses of PEX (12/2011 to 08/2013) and 29 months of treatment with AZA (2 x 50 mg/day, from 01/2012) dysesthesia was still present but not increasing anymore and no further relapses had occurred at last follow-up (06/2014). Follow-up MRI in 02/2013 showed, in addition to the vascular lesions, callosal lesions compatible with focal demyelination and residual discrete T2 hyperintensities in the cervical spinal cord.

Comment: As in 14 further cases, disease started with bilateral ON in this patient. While bilateral ON had traditionally been considered typical for NMO, it was recently shown to be in fact more frequent in AQP4-IgG-negative than in AQP4-IgG-positive patients with NMOSD at disease onset [34]. Of particular interest, LETM was purely sensory, characterized by excruciating dysesthesia, took a protracted or even progressive course, and did not respond to repeat IVMP treatments over a period of 12 months; only PEX and subsequent immunosuppression brought some relief. Surprisingly, 15/19 (78.9 %) patients in this cohort reported at least one occurrence of dysesthesia or pain during acute myelitis attacks. Dysesthesia and pain are also common symptoms in AQP4-IgG-positive NMO and are the only symptom in some patients [34]. Both MOG-IgG- and AQP4-IgG-related EM should be considered in the differential diagnosis of patients presenting with purely sensory symptoms compatible with myelitis.

*Case 10 – Recurrent attacks of ON and myelitis with good partial recovery; subcortical and callosal lesions.* This Asian male patient developed bilateral optic neuritis (VA not documented) in 12/2006 at the age of 35. CSF examination showed a normal cell count and normal protein, glucose, and lactate levels with negative OCB. Brain MRI showed Gd enhancement of both optic nerves, one subcortical lesion in the left cerebral hemisphere, and one lesion in the corpus callosum. Spinal cord MRI was not performed at that time. After IVMP pulse therapy complete remission was documented. Extensive laboratory work-up did not reveal any infectious or rheumatological cause of the symptoms.

A second attack occurred in 10/2007 with clinical symptoms of myelitis (paraparesis, urinary retention, and tingling sensation in the lower extremities). LP revealed an increased cell count (197 cells/μl) with a normal protein level and negative OCB. Spinal MRI showed multifocal myelitis of the upper cervical and thoracic segments (C3-C4, T3-T4) with patchy gadolinium enhancement. After IVMP pulse therapy there was only partial remission and slight unsteadiness of gait remained. MS with an opticospinal focus or neuromyelitis optica was suspected. Treatment with AZA 2 mg/kg (150 mg/day) was initiated and was continued until 07/2008 when treatment was stopped due to clinical stabilization.

The third attack occurred in 03/2010 with unilateral optic neuritis. MRI showed Gd enhancement of the right optic nerve and the previously known T2 lesions with patchy gadolinium enhancement. AQP4 antibodies were tested 4 times during this period and were always negative. Complete remission after IVMP pulse treatment was achieved.

After a fourth attack in 08/2010 with tingling sensation of the lower extremities which was treated with 5 g IVMP with complete remission, treatment with AZA was reinitiated directly after discontinuation of IVMP.

Beginning in 01/2011 (i.e., still within the latency period of AZA) another relapse of myelitis with tingling sensation of the lower extremities and of the right hand (EDSS 2) was treated successfully with IVMP. A VEP examination performed at that time revealed delayed P100 latencies bilaterally with normal amplitudes. OCT showed bilateral thinning of the RFNL. Cerebral and spinal MRI did not show any disease activity. AZA was increased to 200 mg/day and later continued at 150 mg/day. The last MRI of the head and spinal cord was performed in 04/2014 and showed no disease activity. The patient was still relapse-free at last follow-up in 04/2015 with an EDSS of 2.5. In total, this patient has experienced five attacks, comprising two attacks of ON (1 × bilateral) and three of myelitis.

Comment: First, the presence of callosal brain lesions noted in this patient and in others in our study (see cases 6 and 9 here and cases 3 and 8 in part 3 [31]) renders the radiological differential diagnosis of MOG-IgG-positive EM and classical MS more challenging. Callosal lesions have also been observed in AQP4-IgG-positive NMO, where they are typically long (half of the length of the corpus callosum or greater), diffuse, heterogeneous, or edematous [29, 109]. In the present cohort, a longitudinally extensive callosal lesion was noted only in a single patient (see case 3 in part 3 of this series [31]). Second, as a result of ON, RFNL thinning as detected by OCT was present in this as well as in several other patients. Similar findings have previously been reported in AQP4-IgG-positive patients [110, 111]. A detailed OCT analysis of 16 patients with MOG-IgG-positive ON can be found in part 4 of this article series [32].

### III. MOG-IgG-positive recurrent ON

*Case 11 – Three attacks of ON; no brain lesions; functional blindness in the left eye.* A 53-year-old woman first noted reduced vision and pain on moving the right eye in 05/2011. Clinical examination demonstrated a VA of 0.1 in the right eye and 1.0 in the left eye, as well as color desaturation and a relative afferent pupillary defect of the right eye. Cranial MRI showed right optic neuritis, but no parenchymal brain lesions. VEP were slightly delayed in the right eye, but amplitudes were preserved. CSF analysis was normal; in particular, there were no CSF-specific OCB. The patient was treated with high-dose IVMP (2 g/d for 3 days), which resulted in improvement of symptoms. However, about 3 weeks later vision in the right eye deteriorated again.

In 11/2011, she also noted reduced vision in the left eye. She was again administered several courses of IVMP, which did not improve VA, so that she was subsequently treated with four courses of IA. This was associated with almost complete recovery over the next 3 years.

At the end of 11/2014 she again noted reduced VA in the left eye. A cranial MRI performed in 12/2014 showed a left optic neuritis with contrast enhancement as well as hyperintensities in the right optic nerve without contrast enhancement, but no further cerebral lesions. She was again treated with IVMP and four courses of IA, which resulted in only incomplete recovery. In 03/2015, when her VA was finger counting in the left and 0.2 in the right eye, she was admitted to our department. She was tested for serum antibodies to MOG, which were positive both in a live-cell and in a fixed-cell CBA (determined after IA). She was treated with IVMP, five courses of PEX, and rituximab (2 × 1000 mg). However, VA recovered only slightly. At the last follow-up in 05/2015 her vision was 0.2-0.3 in the right and 0.02 in the left eye.

Comment: This case, together with cases 12-18, demonstrates that MOG-IgG-associated ON is not always a monophasic disease but frequently follows a relapsing course. In fact, recurrent ON was noted in 65 % of all patients with isolated ON in the present study. Moreover, the disease took a very severe course with partly therapy-refractory attacks, leading to permanent functional blindness in one eye and substantial loss of VA in the other eye. As in other patients in this series, high-dose and even ultra-high-dose IVMP treatment improved the symptoms only transiently or not at all. Although IA was initially effective, it could not restore vision when used to treat a subsequent relapse. While the reason for that discrepancy is unknown, the relatively long time (6 weeks) between attack onset and IA might have played a role. Alternatively, IA as well as PEX might not result in a sufficient decrease in MOG-IgG titers in some patients if not repeated long enough; finally, other pathomechanisms than antibody-related ones, e.g., T cells, might have been involved.

*Case 12 – Recurrent ON in a child; no brain lesions; severe permanent visual loss.* This Caucasian girl experienced a first episode of ON in 04/2001 at age 6; this attack was bilateral (OD> > OS) with papilledema. VEP showed prolonged P100 latencies. LP demonstrated mild CSF pleocytosis (7 leukocytes/μl). Brain MRI was normal. Symptoms remitted spontaneously. Two years later (04/2003) a second attack of ON occurred, affecting the right eye. Symptoms improved with IVMP. A third attack of ON, again of the right eye, developed 11 months later in 03/2004. CSF was normal. A serum sample drawn in 2004 that was later retrospectively tested was positive for MOG-IgG. When right-sided ON recurred in 08/2008, temporal pallor was noted on fundoscopy bilaterally. In 2009, ocular myositis was suspected, but no additional information is available. In 01/2013 another attack of unilateral ON occurred, associated with contrast enhancement of the right optic nerve; symptoms improved with IVMP. In 04/2013, the patient was started on IFN beta-1a s.c. However, she experienced two more attacks of right-sided ON in 10/2013 and 03/2014, leading to the discontinuation of IFN-beta treatment; both relapses improved after IVMP. MRI still did not show any brain lesions in 10/2013, i.e., 12 years after onset. VA was 0.4 in the right and 0.8 in the left eye at that time. AQP4-IgG antibodies were negative. An OCT examination in 04/2014 showed massive thinning of the RNFL in both eyes (OD > OS). In 07/2014 and again in 01/2015, the patient received two doses of rituximab (2 × 1000 mg, 2 weeks apart). At last follow-up in 03/2015 no further relapses had occurred.

Comment: This is one of the youngest Caucasian patients with MOG-IgG positive ON reported thus far. MOG-IgG autoimmunity is an important differential diagnosis in pediatric patients presenting with NMOSD and other forms of CNS inflammation of unknown cause [107, 112, 113]. The patient’s relapsing disease course confirms that MOG-IgG in pediatric patients with ON is not, as originally thought, limited to monophasic cases [11–13]. Of particular note, the disease has been restricted to the optic nerves in this patient for more than a decade, both clinically and radiologically. As in two other patients (case 6 here and case 4 in part 3 of this series [31]), treatment with IFN-beta was not effective in preventing relapses.

*Case 13 – Recurrent ON with late onset; no inflammatory brain lesions; permanent unilateral blindness.* This Caucasian woman developed severe bilateral optic neuritis in 12/2003 at age 66 following a gastrointestinal infection with positive *Yersinia* serology (species not specified). VA at nadir was 0.1 in both eyes. VEP showed no response of the right eye and was delayed on the left side. Symptoms partly responded to IVMP and oral prednisolone. Brain MRI was normal except for numerous vasculopathic lesions due to decades of hypertension. LP showed CSF pleocytosis (80 cells/μl) but no OCB.

The patient was treated with AZA (150 mg/d) and oral steroids from 03/2004 to 11/2004. However, one ON relapse in the right eye occurred at the end of 04/2004 (with a flare-up in mid-05/2004) and a second in 10/2004 after tapering prednisolone to below 10 mg/day. Symptoms did not substantially improve despite several courses of IVMP (VA 1/35). A follow-up CSF examination performed at that time was normal.

In 11/2004, treatment was switched to MTX (15 mg/week). In 01/2006, the patient experienced another attack of unilateral ON, which led to blindness of the right eye. The patient declined IVMP, and symptoms did not improve spontaneously.

No further relapses have occurred under treatment with MTX since then. In 06/2013 VA of 0 on the right and 1.0 on the left side was documented. At last follow-up, in January 2016, the patient was still on MTX (2.5 mg/day). She never experienced any signs or symptoms of myelitis; spinal MRI (11/2004) was normal. Slightly elevated ANA (1:80) were documented on one occasion, but she has no coexisting autoimmune disorders.

Comment: This case is interesting for several reasons: First, disease started after an acute infection, as also seen in at least 10 other patients in our series. Infections have also been reported as a trigger of acute attacks in AQP4-IgG-positive NMOSD [34] and in classic MS [114]. Second, tapering of steroids resulted in recurrence of the patient’s symptoms. Similarly, withdrawal of steroids or reductions in steroid dosage resulted in flare-ups in 20 further patients in our series. Third, disease resulted in permanent blindness in one eye in this patient. Similarly, a decline in VA ≤0.5 occurred in 85 % of our patients during relapse and severe visual impairment or functional blindness was present in 37 % at last follow-up. This underlines that the notion of MOG-IgG-associated ON being generally mild is incorrect. Fourth, while two attacks occurred under treatment with AZA (and cotreatment with oral steroids) within a period of 9 months in this patient, only one – albeit very severe – relapse occurred under MTX over a period of 10 years. The favorable disease course observed in this and in other MTX-treated patients in this study (see cases 3, 6, and 13 here and cases 6 and 12 in part 3 of this article series [31]) warrants further investigations into the potential efficacy of MTX in MOG-IgG-related autoimmunity.

*Case 14 – Recurrent ON starting at age 28; no brain lesions; permanent functional blindness of the left eye*. This patient presented with unilateral ON (VA OD 1/35) in 05/2009 at age 28. Symptoms improved following high-dose IVMP (3 × 1 g), but recurred shortly thereafter (VA OD 1/35) and only partly responded to a second IVMP (3 × 1 g) cycle (VA 0.2). Brain MRI was normal.

Five months after onset, the patient developed left-sided ON, which fully responded to IVMP. Brain MRI was again normal, except for enhancement of the left optic nerve and edematous thickening of the right optic nerve. VEP examination revealed bilaterally prolonged P100 latencies (OD > OS) and reduced amplitudes.

Another attack of ON, on the left side, occurred 2 months later with severe visual loss (VA 0.1). Fundoscopy revealed mild papilledema. IVMP and 10 plasma exchanges resulted in partial improvement. The patient was started on AZA 100 mg/day in 10/2009 with 6 months cotreatment with oral steroids. In 02/2012, she experienced another relapse of ON, which partially responded to IVMP. The patient had no other immune disorders except for pollinosis. LP had revealed mild pleocytosis (14 cells/μl) but no CSF-restricted OCB and no blood-CSF barrier dysfunction. A serum sample obtained 2 weeks after IVMP therapy was positive for MOG-IgG at a titer of 1:160 in the live-cell assay. MOG-IgG-seropositivity was confirmed in the fixed-cell assay. AQP4-IgG was negative. At last follow-up VA of 0.1 in the left eye was documented.

Comment: As in many patients in this series, the first high-dose IVMP cycle led only to transient remission of symptoms in this case; moreover, only partial recovery was achieved after a second cycle applied for the same attack as well as during two later attacks. The observation of many cases of partial or complete IVMP failure in this series suggests that additional treatment options should be considered in MOG-IgG-positive patients presenting with acute ON or myelitis. It is unknown why IVMP was effective during some attacks, but not all, in this and other patients. However, timing issues and differences in antibody titers, other immunological parameters (e.g., T cell activation), IVMP dosage, and previous or concomitant treatments might all play a role. Note the poor outcome in this case. Functional blindness in at least one eye was also noted in 9 additional patients at last follow-up.

*Case 15 – Three attacks of ON; no brain lesions; full recovery.* This 25-year-old woman presented in 10/2010 with bilateral ON accompanied by visual loss and retro-orbital pain on eye movement. Fundoscopy revealed optic papillitis in both eyes. Except for a marginal difference in biceps tendon reflexes the neurological examination was normal. LP showed normal intracranial pressure, slight pleocytosis (6 cells/μl), normal CSF total protein, glucose, and lactate levels, and negative OCB. VEP were lost in both eyes. Cerebral MRI was normal including the optic nerves. Spinal MRI was not performed at that time. AQP4 antibodies were negative as were extensive infectious and rheumatological laboratory diagnostics. After IVMP therapy (1 g/d for 5 days), the patient completely recovered within 2 weeks.

In 11/2010, 4 weeks after onset of the first symptoms, a second attack occurred with complete visual loss in the right eye and a decrease in VA to 60 % in the left eye. Ophthalmological examination revealed bilateral optic papillitis. VEP in both eyes could not be elicited. IVMP pulse therapy (2 g for 5 days), followed by oral reduction over 4 weeks, led to rapid and complete recovery.

The patient was clinically stable without any maintenance therapy until 11/2014, when she developed a third attack of ON with a decrease in VA of the right eye to 40 % and papillitis of the right optic nerve. The neurological examination was normal. Two cycles of IVMP pulse therapy (1 g/d) for 3 and 5 days, respectively, resulted in complete remission after 2 weeks. In 12/2014 a second brain MRI and a first spinal cord MRI were performed, each with normal findings. SSEP were normal as well.

MOG antibodies were first determined in 03/2015 and were positive. AQP4 antibodies were still negative. At that point the patient was free of symptoms and VEP were normal in both eyes. The patient rejected the idea of commencing any long-term immunosuppressive treatment.

Comment: Despite recurrent disease and severe acute visual impairment with transient blindness and loss of VEP, this patient recovered completely from all attacks following high-dose IVMP (and subsequent oral tapering for one attack). Remarkably, there were no clinical, radiological, or electrophysiological signs of myelitis, brain, brainstem, or cerebellar involvement 3 years after onset; similarly, there was no evidence for spatial dissemination at last follow-up in around a quarter of all MOG-IgG-positive patients in this study.

*Case 16 – Recurrent ON; no brain lesions; significant visual loss in both eyes.* This patient had a first attack of ON at age 58. At last follow-up, 86 months after onset, eight unilateral ON attacks alternately affecting the left and the right eye (never simultaneously bilateral) had occurred but no brain or spinal cord involvement was noted. MOG-IgG were detected retrospectively in a stored sample taken under treatment with oral steroids. VA was 0.4 in the left and 0.5 in the right eye at the last follow-up visit (during remission). There was no relevant comorbidity, including no concomitant autoimmune diseases, and no autoantibodies other than MOG-IgG were detected.

Comment: This case, which is characterized by high disease activity (ARR 1.2) and poor long-term outcome, again illustrates that MOG-IgG-positive ON is not always a monophasic and mild disease. Disease activity varied substantially among untreated patients with isolated ON. While this patient experienced eight ON attacks within just 86 months, no relapse occurred within 72 months in case 22. This renders decisions about long-term treatment difficult, all the more as reliable long-term prognostic markers are lacking. However, with more than 60 % of all patients with isolated ON having developed relapses and around two thirds of those with relapses having been functionally blind or otherwise severely impaired in at least one eye at last follow-up, long-term treatment should be considered in most cases.

*Case 17 - Recurrent bilateral ON; transient unilateral blindness; full recovery.* This Caucasian woman developed a first episode of bilateral ON at the age of 50, presenting with bi-frontal headache, painful eye movements, and blurred vision. VA was 0.5 in the right and 0.8 in the left eye at first clinical presentation. VEP displayed a prolonged P100 latency (right > left). Brain MRI showed contrast agent enhancement of both optic nerves but was otherwise normal, as was spinal cord MRI. CSF revealed mild pleocytosis (21/μl, lymphomonocytic), elevated total protein (929 mg/l), no OCB, and a normal IgG CSF/serum ratio. No other laboratory abnormalities were noted (ANA, ANCA, cardiolipin, beta2-glycoprotein, ACE, soluble IL2R, borreliosis, syphilis). The patient’s medical history was unremarkable except for arterial hypertension. Following high-dose IVMP, VA initially improved to 0.7 right and 1.0 left; however, a bilateral flare-up of ON occurred within 30 days, resulting in a drop of VA to light perception on the right and 0.5 on the left. The patient received five plasma exchanges which resulted in full recovery.

Three months later another bilateral ON attack with a large scotoma occurred. Full remission (bilateral VA 1.0) was achieved with high-dose IVMP. A further 2.5 years later (10/2012) the patient experienced a mild ON attack with blurred vision but without a drop in VA in the right eye. Following the patient’s preferences, no relapse treatment was given and no preventive immunosuppressive treatment was initiated. Re-testing for serum autoantibodies was unremarkable (NMDA-IgA/IgG, amphiphysin, CV2/CRMP5, Ma2/Ta, Ri, Yo, Hu, LGI1, CASPR2, GABA-B receptors, AMPA receptors, GAD, MAG, c-ANCA, p-ANCA, rheumatoid factor, ganglioside antibodies) except for low-titer ANA (1:160). At last follow-up (11/2015) VA was 1.0 in both eyes and no further relapses had occurred.

Comment: Disease again affected exclusively the optic nerves. The excellent long-term outcome despite large scotoma and near-blindness during acute attacks is remarkable. As in around 44 % of all patients, IVMP was only transiently effective when used to treat acute attacks; PEX was required to achieve full remission but, of particular note, could prevent further relapses only for 3 months.

*Case 18 – Recurrent ON with late onset; permanent functional blindness; stabilization under rituximab.* A 58-year-old woman with a history of hepatitis A two decades previously developed amaurosis in the right eye and headache in 04/2014. Clinical examination revealed a VA of 1/100 in the right eye with global alteration of right visual field and delayed and reduced amplitude of P100 wave at visual evoked potential (VEP). Brain MRI showed T2/FLAIR hyperintensity of the right optic nerve with spotty post-contrast enhancement and non-specific subcortical frontal hyperintense lesions. Spinal MRI and CSF were normal. Routine laboratory examinations were normal except for low-titer ANA (1:80). Despite treatment with high-dose IVMP, almost no improvement was achieved (VA 1/10). Two months later (06/2014) the patient developed left-sided ON. Treatment with oral steroids resulted in only mild improvement (5-6/10). Two and four months later (08/2014 and 10/2014), respectively, further ON attacks affecting the left eye occurred; high-dose IVMP was followed by almost full recovery of VA in this eye. At that time, serum positivity to anti-MOG antibodies was detected. In 10/2014 treatment with rituximab (2 × 1000 mg, 2 weeks apart) and oral steroids was started and was followed by further improvement of VA in the left but not in the right eye. At a follow-up visit 1 year after the last attack, VA was 9/10 on the left (with normal VEP) and 1/10 on the right. Of note, CD19 cells were still undetectable 14 months after the first rituximab infusion in this patient.

Comment: While this patient almost completely recovered from three ON attacks in the left eye, no improvement had been achieved after the initial attack, which had affected the right eye and had left the patient functionally blind. This illustrates that the severity of MOG-IgG-associated attacks varies substantially not only between patients but also intraindividually. Studies investigating risk factors for poor attack outcome in MOG-IgG-positive patients are highly warranted.

### IV. MOG-IgG-positive monophasic ON

*Case 19 – Single episode of post-infectious bilateral ON 8 months post partum; no brain lesions; partial recovery.* A 30-year-old woman who was breastfeeding her 8-month-old healthy daughter noticed bilateral blurred vision, pain when moving the eyes, and moderate frontal headaches in February 2011. During the weeks before symptom onset, she had had common colds with mild fever. Her past medical history was otherwise unremarkable. Ophthalmological examination demonstrated reduced VA of the right (1/25) more than of the left eye (0.5), reduced color vision in the right eye, and papillitis of the right more than of the left eye. Visual field examination revealed bilateral large centrocecal scotomas, again more prominent in the right eye. The patient was admitted to our department, where the rest of the neurological examination was normal. Cranial and orbital MRI demonstrated contrast enhancement in both optic nerves, compatible with bilateral optic neuritis, but no lesions in the brain parenchyma. Spinal MRI and chest radiography were unremarkable. CSF analysis revealed a mildly elevated total cell count of 12 white blood cells per μl (reference range, <5/μl) with 88 % lymphocytes and 12 % monocytes. CSF protein and lactate were normal and there were no CSF-specific OCB. A complete blood count and C-reactive protein were normal, as were microbiological (*Borrelia*, *Treponema pallidum*, *Toxoplasma*) and virological tests (herpes simplex virus type 1 and 2, varicella zoster virus). ANA were detectable at a titer of 1:320. Screening for ENA, ANCA, and AQP4-IgG was negative. However, antibodies to MOG were detected in the patient’s serum at a titer of 1:1280. The patient was treated with IVMP (1 g/day for 5 days). An ophthalmological follow-up examination in August 2011 showed markedly improved VA of the right (0.7-0.8) and left (0.7) eye. The visual field defects had almost completely resolved. Funduscopy revealed bilateral mild temporal disk pallor consistent with mild partial optic atrophy.

Comment: This patient developed her first relapse in the third trimester post partum. The first 9 months after pregnancy have been previously identified as a risk period for relapses both in AQP4-IgG-positive NMO [85, 86, 115] and in MS [108]. However, systematic studies comparing ARRs before, during, and after pregnancy in MOG-IgG-positive patients are still lacking. Of note, the temporal association between the two events could still be coincidental in the present case, all the more as disease onset was also preceded by feverish infection. Remarkably, almost complete recovery was achieved despite substantial visual loss during an acute attack.

*Case 20 – Protracted single episode of bilateral ON; delayed SSP; no brain lesions; complete recovery*. This Caucasian man developed a first, bilateral ON at age 70 with severe vision loss (0.5 OD, 0.4 OS), papilledema, scotoma, and delayed P100 latencies (but no or only marginally reduced P100 amplitudes). Treatment with high-dose IV prednisolone (1000 mg 3d) with oral tapering resulted in marked but incomplete short-term recovery with persisting contrast sensitivity impairment and hazy vision. Within 4 weeks from onset of symptoms bilateral visual loss (0.5 OD and OS) and scotoma recurred and improved gradually after a second course of high-dose i.v. prednisolone (1000 mg 3 d) with oral tapering and additional intravenous antibiotics (sobelin, ceftriaxone). Brain MRI revealed slight perineural contrast enhancement around the left optic nerve and signs of ethmoidal cell sinusitis, but was otherwise unremarkable. While spinal MRI did not unequivocally reveal any lesions, tibial nerve SSEP were bilaterally delayed (69 ms on the right; only late components obtainable on the left), suggesting possible subclinical myelitis. Median nerve SSEP were normal except for a side difference in latencies. Transcortical magnetic stimulation to the legs was normal as well. CSF assessment revealed a normal cell profile, mild blood-CSF barrier dysfunction, and no OCB. AQP4-IgG-testing was negative. The patient had been diagnosed with hepatitis B more than 20 years before onset of symptoms; however, Hbs, HBc-IgG, HCV-IgG, and HBE were all negative at the time of ON, as were ANA, ANCA, CRP and rheumatoid factor. Chest radiography did not reveal evidence of sarcoidosis, and serum ACE was normal. At repeated long-term follow-up visits (most recently in 10/2010) complete recovery was confirmed with normal VA and no evidence of scotoma.

Comment: This case, alongside the pediatric cases reported here, illustrates the broad variability in age of onset in MOG-IgG-related disorders. A late onset (>60 years) was also observed in two further patients (66 years in case 13; 64 years in case 9). While MOG-IgG were originally described in pediatric patients with ADEM, these cases demonstrate that MOG-IgG need to be considered also in elderly patients presenting with a first attack of optic neuritis or myelitis. Moreover, this case is one of the few monophasic ones, with no relapse more than 6 years after onset. By contrast, disease followed a relapsing course in 80 % of all MOG-IgG-positive patients in this cohort, irrespective of clinical presentation.

*Case 21 – Single episode of unilateral ON; no brain lesions; complete recovery.* This man presented at age 53 with unilateral retrobulbar ON of the left eye (VA 0/10, peripheral scotoma, intraorbital swelling, and Gd enhancement of the optic nerve with contrast enhancement, VEP delayed and amplitudes reduced; right eye normal). LP was unremarkable with 2 cells/μl, no OCB, and normal CSF/serum ratios of IgG and albumin. MRI revealed no extra-optic nerve brain lesions. MOG-IgG were positive at a titer of 1:1280 prior to treatment. Treatment with high-dose steroids resulted in marked improvement (1 mg/d for 6 days) with complete recovery after some months (VA 10/10). Electrophysiological control examinations 7 and 18 months after onset showed only a residual borderline delay in P100 latency on the left side; brain MRI was still normal 25 months after onset; and at last follow-up at month 75 no new symptoms had occurred. The patient had a history of poliomyelitis during childhood and of brucellosis 12 years prior to ON. Serum AQP4-IgG was absent, but MOG-IgG was present at a titer of 1:1280.

Comment: The favorable attack outcome – with complete recovery following IVMP treatment – and the monophasic disease course – with no new symptoms 75 months after onset – once more illustrate the broad variability in prognosis in MOG-IgG-positive ON.

*Case 22 – Single episode of unilateral ON; no brain lesions; persisting visual deficit.* A 29-year-old man developed sudden loss of vision and reduced visual field in the right eye. VA was initially 20/200 in the affected eye. Unilateral ON was diagnosed. Brain MRI and intracranial pressure were normal. CSF showed normal leukocyte count, IgG index, and protein level, and no OCB. AQP4-IgG was negative. At follow-up, persisting visual loss (20/60) of the right eye was apparent. So far, no further attacks have occurred. Of note, this patient had psychiatric difficulties in the past (classified as ADHD) and a previous history of central diabetes insipidus of unknown cause with onset at age 7 years.

Comment: It remains unknown whether the patient’s diabetes insipidus and psychiatric symptoms were caused by CNS autoimmunity. Of note, however, psychiatric symptoms (psychomotor slowing, disorientation, and impaired consciousness) were present in two further patients in this series (described in detail in part 3 [31]) and occasionally occur also in AQP4-IgG-positive patients [116–118]. Central diabetes insipidus, which is characterized by a lack of antidiuretic hormone (ADH) in the brain, has been previously reported in a patient with NMO but unknown AQP4-IgG and MOG-IgG serostatus [119], and several AQP4-IgG-positive NMOSD patients with Schwartz-Bartter syndrome (also termed syndrome of inappropriate ADH secretion) due to hypothalamic lesions have been described over the past few years [120–122].

*Case 23 – Post-partum episode of ON; possible onset of disease already at age 10; low- rather than high-contrast VA affected.* In 1995, at the age of 10 years, this Caucasian patient had suffered from a self-reported “*Borrelia*-induced meningitis” with headache, diplopia, and bulbar movement pain, with one recurrence 1.5 years later. More than 18 years later, in 07/2015, and 3 months after delivery of her first child, the then 30-year-old woman developed subacute retrobulbar pain and frontal headache on the left side, as well as “focusing deficits” of her left eye. A neurological examination performed 2 weeks after symptom onset was normal including high-contrast VA (1.25 on both sides), but refined vision tests revealed a bilateral reduction of the low-contrast VA (right eye: 0.8; left eye: 0.6). Brain MRI showed a T2-hyperintense left optic nerve lesion and a few bi-frontal non-specific white matter lesions that did not fulfill the diagnostic criteria for MS. MRI of the cervicothoracic spinal cord was unremarkable. LP, performed 3 weeks after symptom onset, revealed slight pleocytosis (9 cells/μl) but was otherwise normal; in particular, OCB were negative. VEP showed normal amplitudes and delayed P100 latencies in the left eye, but only when directly comparing the two eyes with each other (right: 104 ms; left: 112 ms). By contrast, spectral-domain OCT revealed severe bilateral thinning of the RNFL, most prominent in the temporal sectors of both eyes (mean RNFL right: 75 μm, mean RNFL left: 68 μm). Motor, somatosensory, and acoustic evoked potentials were unremarkable. A broad laboratory work-up including ANA, cANCA, pANCA, and AQP4-IgG, was negative. Anti-MOG-IgG antibodies were positive at 1:320. MOG-IgG seropositivity was confirmed by a fixed-cell CBA. While maintaining breastfeeding the patient was treated with IVMP for 5 days (1000 mg once daily), which led to a reduction of the left-sided retrobulbar pain up to the time of the last follow-up in 10/2015. At that time, the patient still refused any long-term immunosuppressant treatment.

Comment: This case is interesting for several reasons. First, the patient presented with unilateral retrobulbar pain and headache but normal VA as detected by Snellen chart and near-normal VEPs. Only additional tests (MRI, OCT, and low-contrast VA testing) revealed marked bilateral optic nerve damage. Similarly, no impairment of high-contrast VA but pathological VEPs were found in cases 25 and 27 here as well as in case 2 in part 3 of this series [31]. These cases indicate that subclinical ON needs to be taken into account also in MOG-IgG-positive patients with apparently normal VA as routinely detected by a Snellen score. Second, symptoms compatible with previous episodes of ON (retrobulbar pain) headache and brainstem encephalitis (diplopia) had occurred 20 and 18.5 years before the present attack in this patient, though it remains unknown whether she was already positive for MOG-IgG at that time. Similarly, disease started with an attack of unilateral ON at age 13 in another patient in this cohort (see case 6 in part 3 of this series [31]), which was followed by a first attack of myelitis only several decades later. Long intervals between first and second attack (up to 17 years) have also been described in AQP4-IgG-positive patients [34]. Third, only OCT, not MRI or VEP, was able to demonstrate damage also of the right optic nerve. Fourth, as in cases 7 and 18 here and in case 12 in part 3 [31], symptoms developed post partum, a period associated with an increased risk for relapse also in MS [108] and in AQP4-IgG-positive NMOSD [115].

*Case 24 – Fifteen attacks of ON with poor response to treatment and unfavorable bilateral functional visual outcome.* In 2013, this 42-year-old female Caucasian patient experienced for the first time typical clinical signs of left-sided ON, with reduced vision, red color desaturation, and eye pain. Fundoscopic examination showed a hyperemic and swollen papilla of the left eye. The VEP amplitudes were reduced on the left side. Further diagnostic work-up including anti-AQP4 antibodies, onconeural antibodies, and immunological screening was negative. The CSF was normal; in particular, there was no pleocytosis, no OCB, and no disruption of the blood-CSF barrier function. MRI of the brain showed an intense, long-segment gadolinium (Gd) enhancement of the left optic nerve, but only few non-MS-specific T2-hyperintense white matter lesions without subclinical progression or Gd enhancement in follow-up scans. The brainstem was never involved clinically or radiologically. Although the patient reported fluctuating paresthesia of both legs and left arm, there was no further clinical, radiological, or electrophysiological evidence for myelitis.

Due to repeated relapses of isolated ON predominately affecting the left eye and partial response to steroid treatment, diagnosis of a chronic relapsing inflammatory optic neuropathy (CRION) was suggested. After 26 months the patient had experienced altogether 15 relapses of ON, consecutively affecting both eyes, but never with simultaneous bilateral involvement.

Except for two attacks (including the initial one), which both remitted completely, she responded only partially to high-dose IVMP treatment. One relapse was treated with PEX, but to no avail. Successive immunosuppressive treatment with AZA (up to 200 mg per day over 9 months and cotreatment with oral steroids), MTX, mycophenolate, and continuous prednisone failed to prevent further attacks of ON. At the time of the last follow-up, 26 months after onset, she had a residual VA of < 0.2 in both eyes.

Comment: The poor visual outcome after just 26 months and the high number of relapses despite regular IVMP treatment for acute relapses and various IS therapies illustrate that MOG-IgG-positive ON may take a severe, relapsing, and sometimes therapy-refractory course. Testing for MOG-IgG should be considered in all patients with suspected CRION.

### V. MOG-IgG-positive recurrent LETM

*Case 25 – Recurrent LETM; slightly delayed VEP; no brain lesions; almost complete recovery.* This 22-year-old Caucasian woman presented with bilateral dys- and hypesthesia of the lower limbs in 11/2011. The neurological examination additionally revealed very mild foot flexor and extensor paresis bilaterally (5-/5) and saddle anesthesia. MRI of the complete neuroaxis (including brain, cervical, thoracic, and lumbar spine) was unremarkable. CSF showed pleocytosis (58 cells/μl) and slightly elevated protein levels (48.9 mg/dl). After negative viral and microbiological diagnostics, initial treatment with acyclovir and ceftriaxone was changed to 4 × 1 g IVMP and, subsequently, oral steroids with tapered dose reduction. Clinical symptoms receded to slight plantar dysesthesia with good response to pregabalin.

In 03/2012 a relapse with bilateral dys- and hypesthesia occurred, this time affecting the upper limbs (fingertips bilaterally), lower limbs (thighs), and trunk (T4). Cerebral MRI again showed no pathologies; spinal MRI, however, revealed two LETM lesions, one stretching from C1 to C4, the other from C7 to T9; both showed swelling, the latter also Gd enhancement. Diagnostic workup again revealed pleocytosis in the CSF (25 cells/μl) and elevated CSF protein levels (53.2 mg/dl). OCB and AQP4-IgG were negative. Serologically, TPO antibodies were positive, but otherwise there was no indication for another autoimmune disease or vasculitis. Electrophysiology showed marginal P100 delay (117 – 118 ms bilaterally) in VEP and reduced amplitudes in SSEP (pronounced on the right). Again, 4 × 1 g IVMP with tapered dose reduction was applied. With the suspected diagnosis of AQP4-IgG-negative NMO, AZA was started at the end of 03/2012. The neurological symptoms improved under this therapy but the patient developed recurrent genital condyloma, necessitating operative removal.

The patient presented at our hospital for the first time in 11/2014. She had symptoms of slight gait ataxia, mainly at night, slight hypesthesia (inner thighs), and complained of severe fatigue, partly accentuated due to the recurrent operative procedures during the past few weeks. Thus the decision was made to switch the medication to rituximab, and AZA treatment was ended in 12/2014. During the drug-free interval of 3 months, the patient noted a remarkable clinical amelioration of the neurological and neuropsychological symptoms and finally came to be very reserved concerning the initiation of rituximab. Due to the patient´s concerns, it was decided to continue with close clinical and MRI follow-up. At the last two follow-up visits (03/2015 and 06/2015) the patient showed continuous recovery with only slight residual nocturnal gait ataxia (EDSS 1). MRI showed no new lesions and no Gd enhancement. At the first follow-up visit, MOG-IgG were found using a commercial CBA (Euroimmun) and were confirmed in the live-cell CBA.

Comment: The good long-term outcome (EDSS 1.0 at 3.5 years after onset) despite three myelitis attacks and despite the presence of extensive inflammation affecting the spinal cord over a length of 14 vertebral segments is remarkable. MOG-IgG-positive myelitis was longitudinally extensive in most of our patients and involved large parts of or even the entire spinal cord in some cases. Purely sensory attacks, as observed here, occurred in 13 other cases and, as said before, dysesthesia and pain were common symptoms in this cohort. Of note, good response to pregabalin was noted in this patient.

*Case 26 – Recurrent LETM; spinal cord biopsy; partial recovery.* This Caucasian man developed a first attack of myelitis in 12/2013 at age 41, with tetraparesis. Spinal cord MRI revealed an LETM lesion extending from C3 to C5. Brain MRI showed a right-sided Gd-enhancing T2 lesion adjacent to the posterior horn of the right ventricle. LP demonstrated mild pleocytosis (23 cells/μl) but no OCB. A decompression operation was performed in 01/2014 due to a suspected diagnosis of cervical myelopathy. However, symptoms worsened again in 04/2014 and a biopsy sample was analyzed to rule out neoplasm and vasculitis. Neuropathology revealed T cell infiltration but there was no specific staining for IgG and complement deposition. Follow-up MRI examinations over a period of 1 year persistently showed contrast enhancement in the cervical spinal cord. Two cycles of high-dose IVMP in 10/2014 and 11/2014 with subsequent oral steroid therapy resulted in only transient improvement. Several tests for AQP4-IgG were negative. In 02/2015 MOG-IgG was tested for the first time and was positive at low titer (1:160) in a live-cell assay; the result was confirmed in a commercial fixed-cell assay for MOG-IgG (Euroimmun). Broad differential diagnosis for infectious, (para)neoplastic, and autoimmune conditions was unremarkable. At follow-up in 02/2015 residual paresis (EDSS 4) was present; MOG-IgG were again positive at a titer of 1:160 and were confirmed in a second, commercial fixed-cell CBA (Euroimmun). There was no clinical, MRI, or electrophysiological (normal VEP 03/2015) evidence of optic nerve involvement. In September 2015, just five months after the first infusion of rituximab, the patient developed a relapse of acute myelitis with severe paresis. PEX resulted in only partial recovery. At last follow-up in March 2016, an EDSS of 6 was noted.

Comment: The differential diagnosis of LETM lesions include, among others, tumors, lymphoma, and spinal cord compression. Accordingly, reports on (unnecessary) neurosurgical procedures, including biopsies, exist also in AQP4-IgG-positive LETM patients [123–126]. Except in the case of emergency, AQP4-IgG and MOG-IgG should be excluded using at least two sensitive assays, at least one of which should be a cell-based assay [8, 29, 124], before any such procedure is considered.

### VI. MOG-IgG-positive monophasic LETM

*Case 27 – Single episode of LETM; no brain lesions; partial recovery at discharge.* A 23-year-old Caucasian man presented with a sensory level at T4, local dysesthesia, mildly positive Babinski reaction, and bladder and erectile dysfunction shortly after an unspecified infection. MRI showed an LETM lesion extending from C3 to C7 with swelling. Brain MRI was normal. LP revealed lymphomonocytic pleocytosis (59 cell/μl) and mild blood-CSF barrier dysfunction (QAlb 6.96), but no intrathecal IgG synthesis (no OCB, QIgG normal). Further examinations for infection (*Borrelia burgdorferi*, *Treponema pallidum*, HAV, HBV, HCV, HIV, CMV, EBV, HSV1, HSV2, FSME, *Mycoplasma pneumoniae*) or common autoimmune disorders (ANA, ANCA, rheumatoid factor, CRP, C3d) were negative except for slightly elevated phospholipid/glycoprotein beta2 IgG antibodies (IgM negative). VEP were bilaterally delayed, indicating a history of subclinical ON; MRI of the orbit was unrevealing. MOG-IgG were positive at a titer of 1:10,240. Treatment with rocephine, acyclovir and, subsequently, high-dose steroids and oral tapering was followed by marked improvement. At discharge, residual mild and circumscript paresthesia as well as bladder dysfunction (requiring urinary catheterization) was present.

Comment: In this and two other cases without a history of clinically apparent ON (case 25 here and case 2 in part 3 of this series [31]), delayed P100 latencies were noted, suggesting subclinical optic nerve inflammation. The predictive value of a positive VEP for a future clinical ON relapse in patients with MOG-IgG-positive myelitis is so far unknown. Of note, the current diagnostic criteria for AQP4-IgG-negative NMOSD do not take into account VEP results but only clinical episodes of ON [29].

### VII. Postvaccinal ON and myelitis

*Case 28 – Recurrent myelitis and ON after vaccination against tetanus, diphtheria and pertussis resulting in functional blindness and tetraparesis; poor outcome.* A 47-year-old Caucasian woman presented with acute sensorimotor tetraparesis (upper limbs: BMRC grade 4; lower limbs: BMRC grade 0), transient somnolence, and respiratory distress. Symptoms had started 12 days after vaccination against tetanus, diphtheria, and pertussis (Boostrix®) and had been preceded by a 2- to 3-day episode of fever prior to symptom onset. MRI showed a single parieto-occipital lesion and a longitudinally extensive spinal cord lesion extending over 15 vertebral segments (C2 to T9). CSF examination revealed moderate pleocytosis (210 white cells/μl) and disturbed blood-CSF barrier function but no CSF-restricted OCB. The symptoms responded only partially to IVMP and IA. As MOG-IgG was positive, treatment with rituximab was started. By 11 days after the first infusion, spinal cord T2 hyperintensities had resolved almost completely. However, just 7 weeks after the second infusion of rituximab and 3 months after onset of the first attack, the patient developed an episode of simultaneous myelitis and unilateral optic neuritis leading to severe loss of vision in the right eye. P100 latencies were delayed in both eyes and amplitudes reduced in the right eye. Brain MRI demonstrated an increase in size of the parieto-occipital lesion. Spinal MRI showed T2 hyperintensities from C7 to T8, predominantly in the posterior columns. Treatment with IVMP and IA was followed by incomplete remission of the symptoms. Just 48 days after onset of the second attack, the patient was re-admitted with a new attack of unilateral ON in the left eye resulting in almost complete visual loss (VA 0.05). Treatment with IVMP, IA, and cyclophosphamide led only to partial recovery (VA 0.16 at discharge). At last follow-up, severe spastic paralysis of the lower limbs and an EDSS score of 8 was documented.

Comment: Disease onset in this patient followed vaccination with a polyvalent vaccine against tetanus, diphtheria, and pertussis. Although a causal link between the two events cannot be proved, the close temporal association is highly suggestive of vaccine-mediated immune activation. Of particular note, symptoms also started within 2 weeks after a polyvalent vaccination against tetanus, diphtheria, and pertussis (as well as polio and influenza virus) in a second MOG-IgG-positive patient of this cohort (see case 8 in part 3 [120]). Whether molecular mimicry between vaccine epitopes and neural antigens played a role or whether vaccination only indirectly triggered or promoted the immune reaction against MOG is currently unknown but certainly warrants further investigation.

## Abbreviations

ADEM: acute disseminated encephalomyelitis
AQP4: aquaporin-4
ARR: annualized relapse rate
AZA: azathioprine
BCSFB: blood-CSF barrier
BMRC: British Medical Research Council
CRION: chronic relapsing idiopathic optic neuropathy
CSF: cerebrospinal fluid
EDSS: expanded disability status scale
EM: encephalomyelitis
EP: evoked potentials
GLAT: glatiramer acetate
IA: immunoadsorption
IFN-beta: interferon-beta
IgG: immunoglobulin G
IM: immunomodulatory
IS: immunosuppressive
IVIG: intravenous immunoglobulins
IVMP: intravenous methylprednisolone
JCV: John Cunningham virus
LEON: longitudinally extensive optic neuritis
LETM: longitudinally extensive transverse myelitis
LP: lumbat puncture
MOG: myelin oligodendrocyte glycoprotein
MRI: magnetic resonance imaging
MS: multiple sclerosis
MTX: methotrexate
NAT: natalizumab
NMO: neuromyelitis optica
NMOSD: neuromyelitis optica spectrum disorder
NETM: non-longitudinally extensive transverse myelitis
OCB: oligoclonal bands
OCT: optical coherence tomography
ON: optic neuritis
QAlb: albumin CSF/serum quotient
QIgG: IgG CSF/serum quotient
RA: rheumatoid arthritis
SSEP: somatosensory evoked potentials
VA: visual acuity
VEP: visual evoked potentials
VS: vertebral segment
WCC: white cell count
